# New insight into the eco-friendly synthesis of MgO NPs using olive pomace extract: combined experimental and DFT study for Congo red adsorption

**DOI:** 10.1039/d6ra02871c

**Published:** 2026-07-31

**Authors:** Sabrina Mechati, Meriem Zamouche, Hichem Tahraoui, Zakaria Laggoun, Oumnia Rayane Benkouachi, Nasreddine Hadjadj, Nora Cherb, Sonia Cherif, Mohammed Kebir, Mouni Lotfi, Amine Aymen Assadi, Ahmed Amine Azzaz, Abdelatif Amrane

**Affiliations:** a Laboratoire de Recherche sur le Médicament et le Développement Durable (ReMeDD), Department of Environmental Engineering, Salah Boubnider University of Constantine 3 Constantine Algeria Meriem.zamouche@univ-constantine3.dz; b Laboratory of Biomaterials and Transport Phenomena (LBMTP), University Yahia Fares Medea 26000 Algeria; c Laboratory of Reaction Engineering, Department of Mechanical and Process Engineering, 14 University of Science and Technology Houari Boumediene (USTHB) Algiers-Bab Ezzouar 16111 Algeria; d Univ Rennes, Ecole Nationale Supérieure de Chimie de Rennes, CNRS, ISCR (Institut des 17 Sciences Chimiques de Rennes) –UMR 6226, Univ Rennes F-35000 Rennes France; e Department of Environmental Engineering, University Salah Boubnider- Constantine 3 New City Ali Mendjeli Constantine 25000 Algeria; f Department of Process Engineering, Faculty of Technology, Ferhat Abbas University Sétif Algeria; g Research Unit Environmental Chemistry and Structural Molecular CHEMS, Université Frères Mentouri Constantine 1 Constantine Algeria; h Biotechnology Research Center (CRBt), Division of Biotechnology and Environment Algeria; i Laboratory of Reaction Engineering, Faculty of Mechanical Engineering and Process Engineering USTHB BP 32 Al Alia 16111 Algiers Algeria; j Scientific and Technical Center of Research in Physical and Chemical Analysis CRAPC BP 384 Bou-Ismail 42004 Algeria; k Laboratoire de Gestion et Valorisation des Ressources Naturelles et Assurance Qualité, Faculté SNVST, Université de Bouira 10000 Bouira Algeria; l College of Engineering, Imam Mohammad Ibn Saud Islamic University (IMSIU) 11432 Riyadh Saudi Arabia; m UniLaSalle, ECLORE, ULR 7519, Campus de Ker Lann 35 170 Bruz France

## Abstract

In this study, magnesium oxide nanoparticles (MgO NPs) were synthesized *via* a green method using olive pomace extract as a natural reducing and stabilizing agent. XRD, FTIR, SEM-EDX, TG/DTG, and BET analyses confirmed the formation of crystalline, quasi-spherical MgO with a cubic structure and an average crystallite size of ∼13 nm. The MgO NPs were applied for the removal of Congo Red (CR) dye from aqueous solutions, achieving 91.5% removal within 30 min and over 95% under optimal conditions (pH ≈ 7, dose = 0.2 g, *C*_0_ = 20 mg L^−1^, *T* = 298 K). Equilibrium data fitted the Dubinin–Radushkevich isotherm, and kinetics followed a pseudo-second-order model, indicating chemisorption as the dominant mechanism. Thermodynamic parameters indicated spontaneous (Δ*G*° = −31.16 to −31.43 kJ mol^−1^) and exothermic (Δ*H*° = −27.21 kJ mol^−1^) adsorption, with increased disorder at the solid–liquid interface (Δ*S*° = +13.50 J (mol^−1^ K^−1^)) and a slight decrease in adsorption at higher temperatures. DFT and MD studies provided molecular-level insights, showing strong electrostatic and charge-transfer interactions between CR sulfonate groups and Mg^2+^ sites, supported by a low HOMO–LUMO gap (∼2.83 eV) and favorable adsorption configurations. These results demonstrate that green-synthesized MgO NPs from olive pomace are efficient and environmentally friendly adsorbents for dye removal from wastewater.

## Abstract 2

Green-synthesized MgO nanoparticles derived from olive pomace exhibited excellent adsorption performance for Congo Red removal, highlighting a sustainable and eco-friendly wastewater treatment approach.

## Graphical abstract

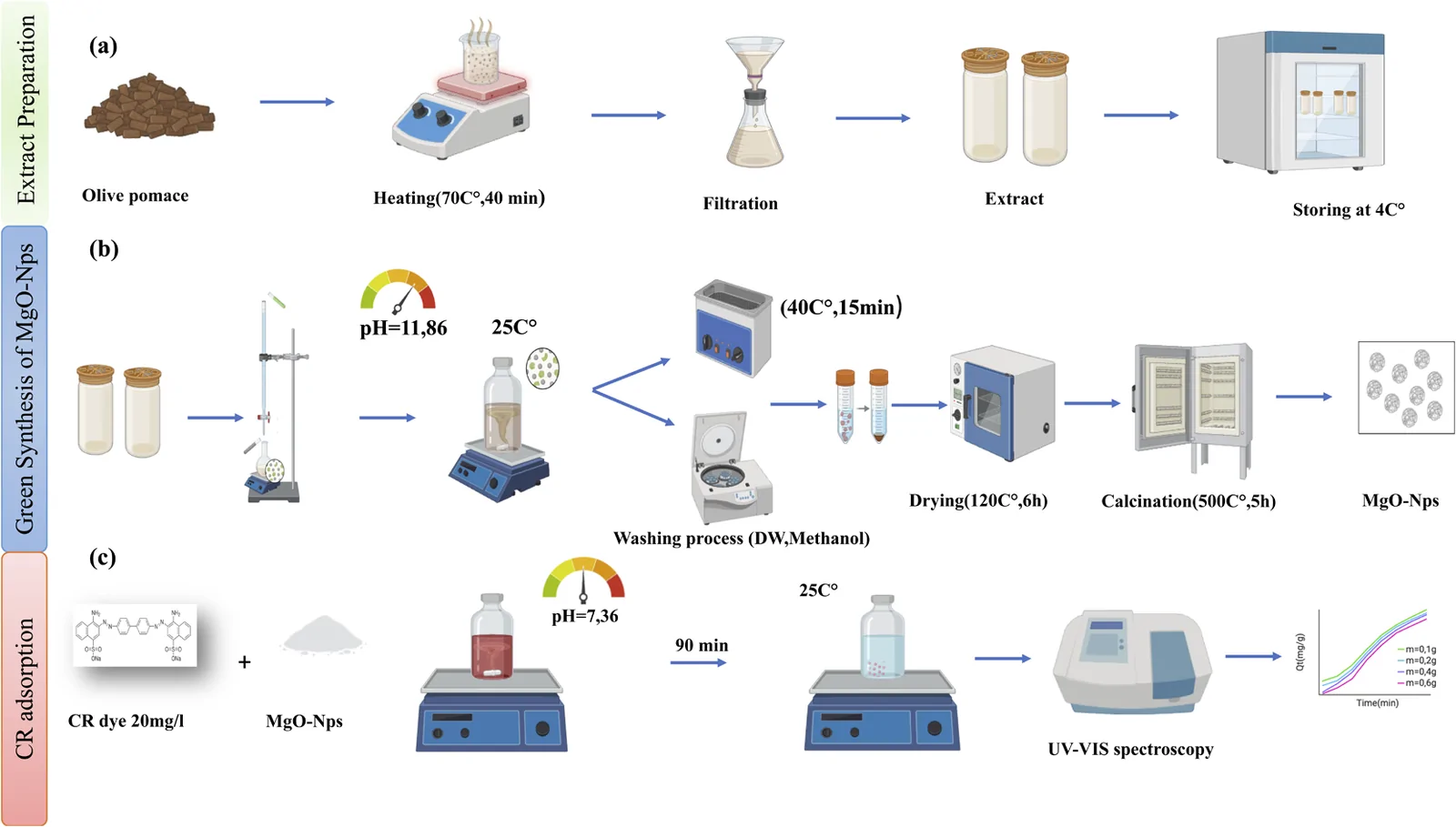

## 1. Introduction

Environmental pollution, particularly in aquatic ecosystems, has emerged as a critical global challenge driven by rapid industrialization, population growth, and increasing demand for resources.^1–4^ Industrial sectors such as textile, pharmaceutical, and chemical manufacturing discharge significant volumes of untreated or inadequately treated effluents, leading to severe ecological degradation and posing risks to human health.^5,6^ This situation is particularly alarming in developing countries, where water resources are already under significant stress due to both overexploitation and contamination.^7–9^

Despite accounting for approximately 22% of global freshwater consumption, industrial activities are responsible for releasing a substantial proportion of wastewater without proper treatment, exacerbating water scarcity and environmental stress.^2,8^ In this context, the removal of toxic contaminants from wastewater remains a major environmental challenge, and conventional treatment methods are often insufficient to efficiently eliminate persistent pollutants.^10,11^

Among industrial pollutants, synthetic dyes represent a major concern due to their complex aromatic structures, high chemical stability, and resistance to biodegradation. These compounds not only pose toxic, carcinogenic and mutagenic risks but also disrupt aquatic ecosystems by reducing light penetration and dissolved oxygen levels.^12,13^ Congo Red (CR), a widely used anionic azo dye, is particularly problematic due to its persistence and hazardous nature, making its removal from wastewater a priority.^14^

Various treatment technologies, including membrane filtration, coagulation, biological processes, and advanced oxidation methods, have been developed for dye removal.^12,15,16^ Although these techniques are effective under certain conditions, they often present several limitations such as high operational costs, energy consumption, generation of secondary pollutants, and limited removal efficiency. However, many of these approaches suffer from limitations such as high operational costs, secondary pollution, or limited efficiency. However, many of these approaches suffer from limitations such as high operational costs, secondary pollution, or limited efficiency. In this context, adsorption has gained significant attention as a simple, cost-effective, and efficient technique for removing persistent organic pollutants (POPs), particularly dyes and phenolic compounds.^16,17^

Recently, nanostructured materials, particularly magnesium oxide nanoparticles (MgO NPs), have attracted considerable interest due to their high surface area, tunable surface properties, and strong adsorption capacity.^18,19^ However, conventional synthesis methods of MgO nanoparticles typically involve toxic reagents, harsh conditions, and high energy consumption, which limit their environmental sustainability.^20,21^

To address these limitations, green synthesis approaches have emerged as a promising alternative, utilizing natural resources such as plant extracts to produce nanoparticles in an eco-friendly manner. Plant-derived biomolecules, including polyphenols and flavonoids, act as reducing and stabilizing agents, eliminating the need for toxic chemicals while enhancing the functional properties of the synthesized materials.^20,22–24^ This green route provides a sustainable, low-cost, and environmentally benign pathway for nanoparticle synthesis.

In this context, agricultural waste valorization represents a highly sustainable strategy within a circular bioeconomy. Olive pomace, the primary solid lignocellulosic by-product of the olive oil extraction industry, accounts for approximately 35–40% of the total mass of processed olives. This heterogeneous biomass is exceptionally rich in structural biopolymers (cellulose, hemicellulose, and lignin) as well as highly active bioactive compounds, including long-chain polyphenols and flavonoids. Due to their potent antioxidant and intrinsic reducing/stabilizing capacity, these plant-derived biomolecules can effectively eliminate the requirement for hazardous chemical reductants during nanostructure synthesis. Despite its clear ecological advantages and abundant availability, the application of this specific biomass for the green synthesis of optimized MgO nanoparticles aimed at industrial dye remediation remains insufficiently explored.

Therefore, the main idea of this work is to propose a green and sustainable synthesis route for MgO nanoparticles using olive pomace extract as a natural reducing and stabilizing agent. This approach aims to combine waste valorization with the development of efficient adsorbent materials for environmental remediation. The synthesized nanoparticles were thoroughly characterized using XRD, SEM, FTIR, TGA, and BET analyses. The adsorption behavior was investigated through isotherm, kinetic, and thermodynamic studies. Furthermore, Density Functional Theory (DFT) calculations were employed to provide a molecular-level understanding of the adsorption mechanism.^25,26^

The advantages of the proposed approach include the elimination of toxic chemicals, reduction of environmental impact, valorization of agricultural waste, and the production of efficient and sustainable nanomaterials with high adsorption performance. Compared with conventional MgO synthesis routes, this integrated experimental and theoretical approach provides new insights into the design of sustainable nanomaterials for wastewater treatment applications and highlights the added value of coupling green chemistry with advanced modeling tools.

## 2. Experiments and methodology

### 2.1. Chemical reagents

Analytical-grade reagents were used without further purification, including magnesium nitrate hexahydrate (Mg(NO_3_)_2_·6H_2_O; 99.99%), sodium chloride (NaCl), Congo Red (C_32_H_22_N_6_Na_2_O_6_S_2_), and methanol (CH_3_OH>99%). All chemicals were purchased from Sigma-Aldrich. Distilled water was used throughout all experiments.

### 2.2. Olive pomace collection and preparation

Olive pomace extract was used as a green reducing and stabilizing agent due to its richness in phenolic compounds and other phytochemicals, which can act as natural reducing and capping agents, promoting nanoparticle formation in an environmentally friendly manner. Olive pomace was collected from a traditional oil mill located in Béjaïa, Algeria. It was thoroughly washed with running water to remove residual oil, dust, and soluble impurities, then rinsed with distilled water and air-dried for 48 hours. The dried material was subsequently ground and sieved to obtain a uniform particle size of 200 µm.

### 2.3. Green synthesis of MgO nanoparticles (MgO NPs)

#### 2.3.1. Preparation of olive pomace extract

To extract bioactive phytochemicals, dried olive pomace powder was mixed with distilled water at a solid-to-liquid ratio of 10% (w/v) and heated at 70 °C for 30 min under continuous stirring. The mixture was then allowed to cool to room temperature and filtered using Whatman No. 1 filter paper. The obtained extract was stored at 4 °C in an amber glass bottle until further use.^27–30^

#### 2.3.2. Synthesis of MgO NPs

MgO nanoparticles were synthesized *via* a green-assisted precipitation method using olive pomace extract and an aqueous magnesium nitrate solution. While previous studies^30–33^ guided the general principles of green synthesis, the exact protocol, including the extract/metal salt ratio, pH adjustment, stirring, ultrasonic treatment, and calcination conditions, was systematically optimized in our laboratory to achieve reproducible, stable, and crystalline MgO nanoparticles.

In brief, 20% (v/v) olive pomace extract was mixed with 80% (v/v) of a 0.1 M magnesium nitrate solution. The mixture was stirred at 350 rpm for 30 min. The pH was then adjusted to ∼11.8 by the gradual addition of a dilute alkaline solution, leading to the formation of Mg(OH)_2_ precipitate. The reaction mixture was maintained at room temperature for 24 h to ensure complete precipitation and stabilization.

To facilitate particle formation and enhance dispersion, the obtained precipitate was subjected to ultrasonic treatment (Nahita U30) at 40 °C for 20 min. The precipitate was then centrifuged (Dlab DM0412) at 4500 rpm for 20 min and washed several times with a methanol–water mixture to remove residual impurities [28]. The purified precipitate was dried at 120 °C for 6 h in a vacuum oven (Memmert UN30) and finally calcined at 600 °C for 5 h to yield crystalline MgO nanoparticles.

The formation of MgO nanoparticles occurs in two main steps. First, magnesium nitrate reacts with the alkaline solution to form magnesium hydroxide, as represented in eqn (1):^34,35^

1 Mg(NO_3_)_2_ + 2 NaOH → Mg(OH)_2_ + 2 NaNO_3_

Then, the magnesium hydroxide is converted into magnesium oxide upon calcination, as shown in eqn (2):^34,35^

2 Mg(OH)_2_ → MgO + H_2_O

**Fig. 1:**
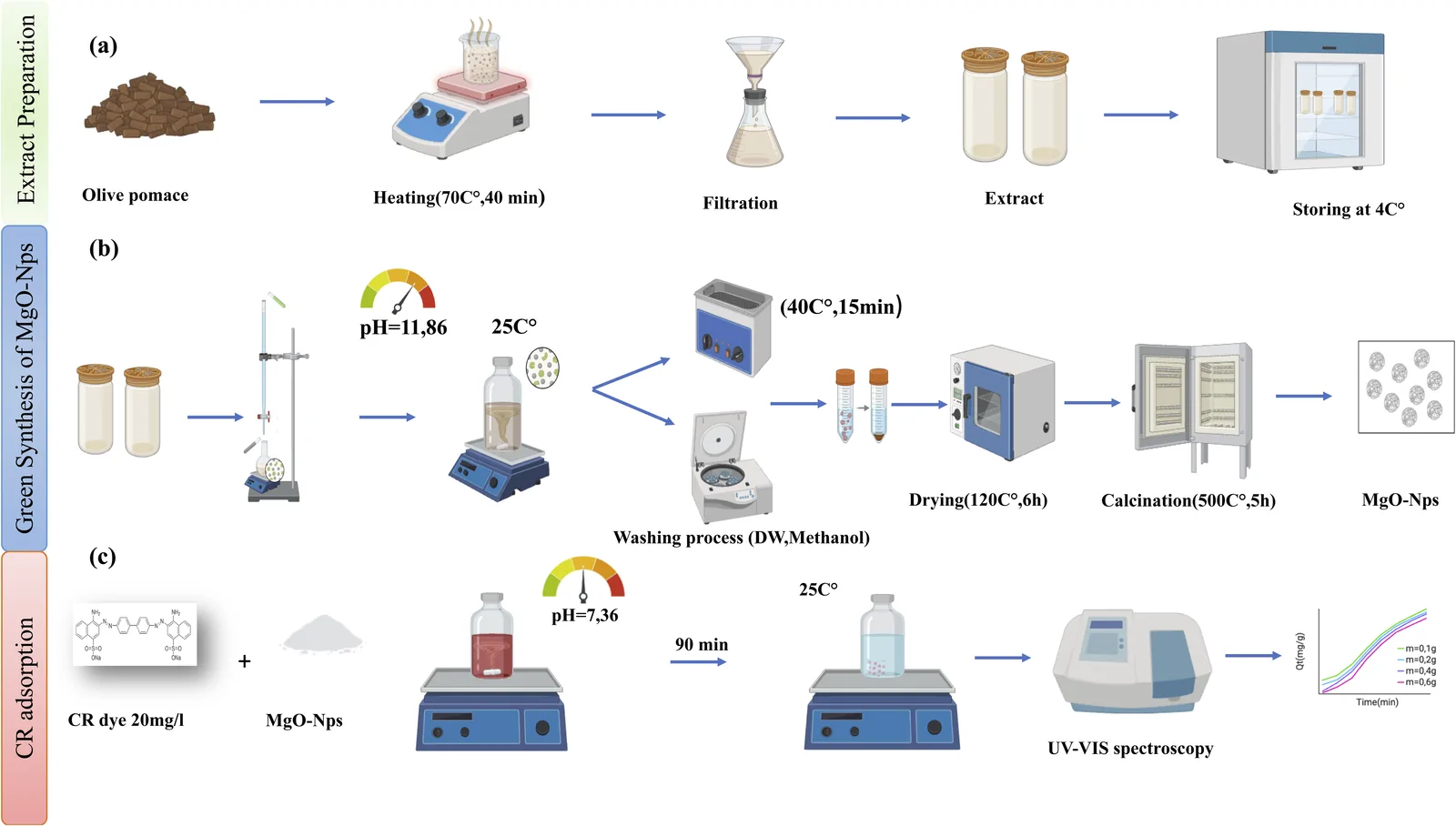
Schematic representation of the green synthesis of MgO NPs: (a) preparation of aqueous extract from olive pomace, (b) green synthesis of MgO NPs, and (c) Congo Red adsorption process.

These reactions represent the standard chemical pathway, while the exact sequence and conditions of this synthesis were optimized in our lab to ensure reproducibility, particle stability, and high crystallinity. A schematic of the synthesis process is presented in Fig. 1.

### 2.4. Congo red adsorption studies

For adsorption experiments, 0.2 g of calcined MgO NPs was added to 200 mL of a 20 mg L^−1^ Congo Red solution at room temperature. The mixture was stirred at 350 rpm, and aliquots were collected at predetermined intervals and centrifuged at 4500 rpm for 6 minutes. The residual dye concentration was measured using UV-Vis spectrophotometry at *λ*_max_ = 497 nm.

The adsorption capacity (*q*_e_) and removal efficiency (*R*%) were calculated as follows:^36–38^3
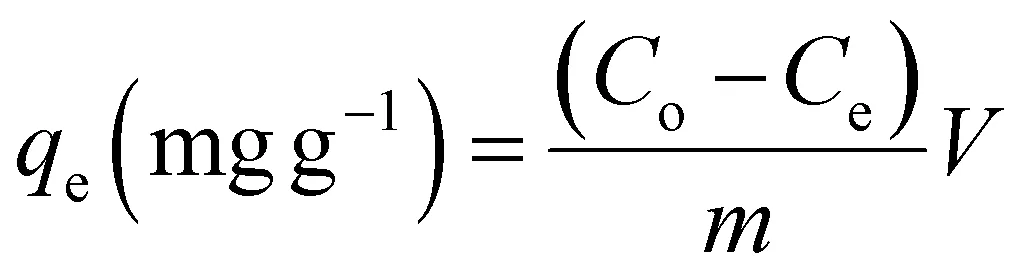
4
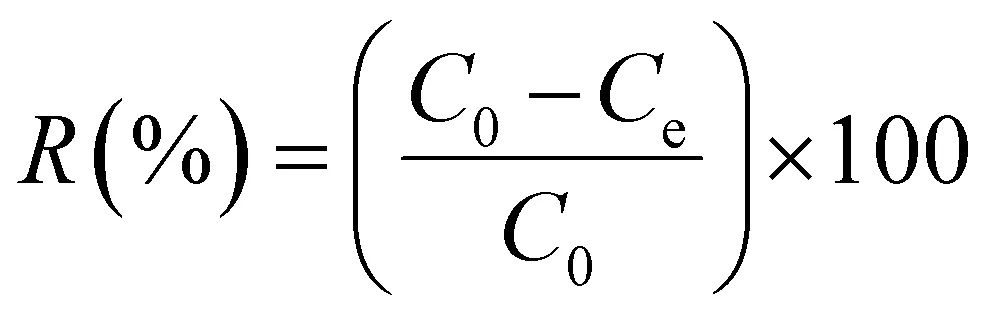
where *C*_0_ and *C*_e_ present the initial and equilibrium dye concentrations (mg L^−1^), *V* is the solution volume (L) and *m* is the mass of MgO NPs used (g).

Kinetic studies were performed over 90 minutes, with pH varied from 5 to 11, initial Congo Red concentrations from 20 to 100 mg L^−1^, and temperatures of 20, 40, and 60 °C.

Structural and phase characterizations were conducted *via* X-ray diffraction utilizing a Bruker D8 Advance diffractometer equipped with a Cu-Kα radiation source (*λ* = 0.15406 nm) scanned with a step sizing of 0.02°. FTIR spectral data were logged on a Nicolet iS50 spectrometer configured at a resolution accuracy of 4 cm^−1^ across 32 continuous scans. Thermal degradation monitoring was recorded utilizing a Mettler Toledo TGA/DSC 3+ thermal analyzer swept under an inert nitrogen ambient stream at a fixed heating rate of 10°C min^−1^ up to 800 °C. Morphological features and elemental distributions were cross-verified *via* SEM-EDX and textural parameters *via* BET evaluation. The point of zero charge (pH_PZC_) was determined using the pH drift method^39^,^40^ to evaluate surface charge properties affecting Congo Red adsorption.

### 2.5. Error calculation on kinetic model parameters

The performance of the adsorption kinetic models was evaluated using the sum of squared errors (SSE) and the root mean squared error (RMSE), calculated as shown in Table 1.

**Table 1:** Mathematical expressions for error calculations of kinetic model parameters

Error	Mathematical equation	Reference/Notes
SSE	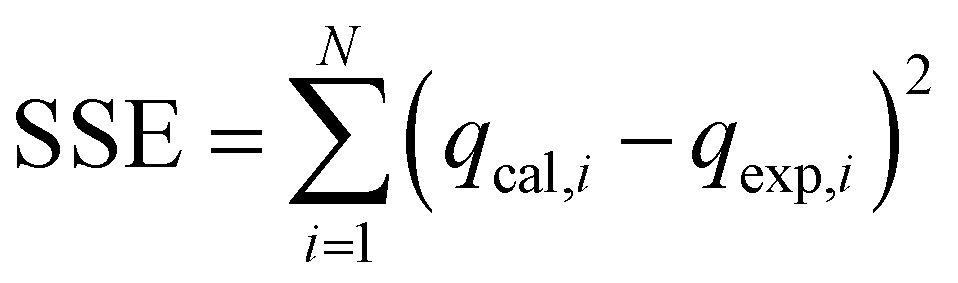	41/(5)
RMSE	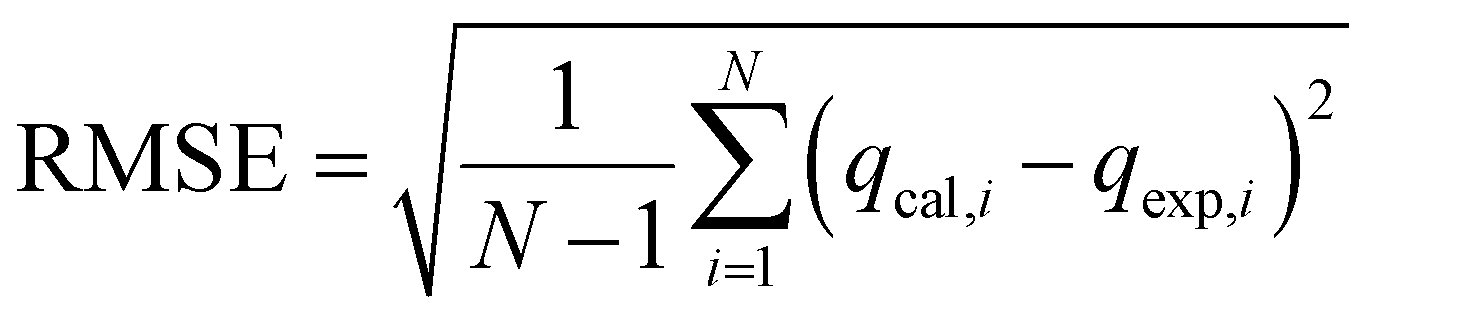	41/(6)

Where, *q*_exp_ (mg g^−1^) and *q*_cal_ (mg g^−1^) represent the experimentally determined and calculated equilibrium adsorption capacities, respectively, and *N* is the total number of data points used in the error calculations.

### 2.6. Reusability

The reusability of MgO NPs was evaluated over three adsorption–desorption cycles. After each cycle, the nanoparticles were recovered by filtration, washed with methanol to desorb the dye, rinsed with distilled water, and calcined at 500 °C for 3 h to regenerate the active surface. The residual adsorption capacity after each cycle was compared to that of the fresh MgO NPs to assess potential loss of activity and the practical suitability of the material for repeated use in wastewater treatment.

## 3. Results and discussion

### 3.1. Characterizations

#### 3.1.1. XRD analysis for adsorbent

**Fig. 2:**
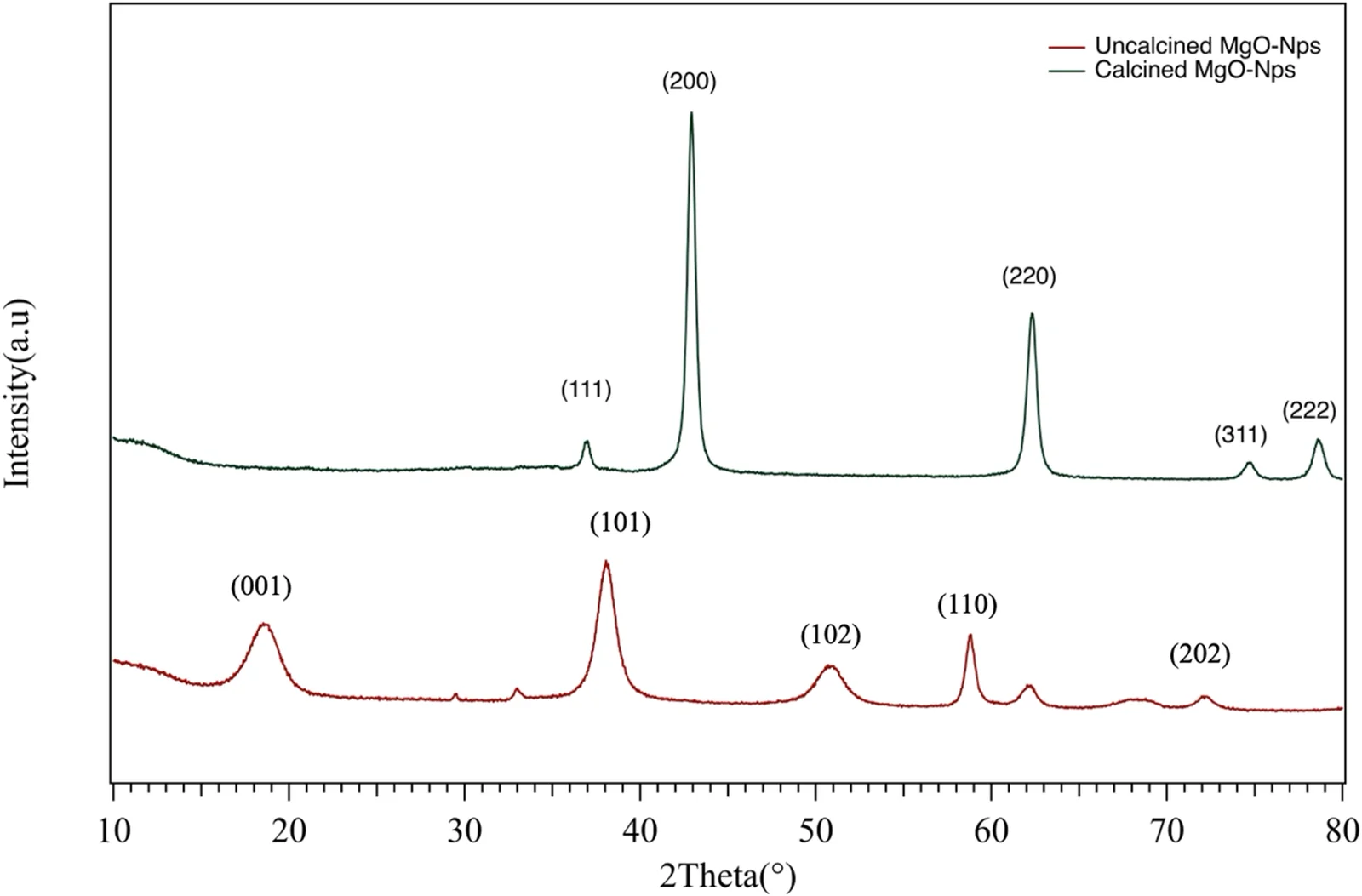
XRD patterns of uncalcined and calcined MgO NPs.

The crystalline structure of the synthesized material before and after calcination was investigated using X-ray diffraction (XRD) in the 2*θ* range of 10–80°. The diffraction pattern of the uncalcined sample (Fig. 2) exhibits relatively broad and low-intensity peaks with a diffuse background, indicating a low degree of crystallinity and the presence of precursor phases. A diffraction peak observed around 18–19° (2*θ*) can be attributed to the (001) reflection of magnesium hydroxide, Mg(OH)_2_ (brucite), suggesting the formation of an intermediate phase during the synthesis process. Such a phase is commonly formed during precipitation-based or green synthesis processes prior to thermal treatment. The peak broadening indicates that the material consists of poorly crystallized hydroxide phases or partially amorphous intermediates, possibly containing residual organic species originating from the olive pomace extract used during the synthesis process.

After calcination, the XRD pattern shows sharp and intense diffraction peaks, indicating a significant increase in crystallinity and the formation of a well-defined crystalline phase. The main diffraction peaks are located at approximately 36.9°, 42.9°, 62.3°, 74.7°, and 78.6° (2*θ*), corresponding to the (111), (200), (220), (311), and (222) crystallographic planes, respectively. These peaks are characteristic of magnesium oxide (MgO) with a cubic crystal structure (space group Fm-3m) and are in good agreement with the standard diffraction data for MgO nanoparticles (JCPDS card No. 45–0946). The absence of additional peaks confirms the high purity of the calcined sample. Similar diffraction peak positions have been reported in several studies on MgO nanomaterials synthesized *via* chemical or green routes.^42–44^

The transformation observed between the two diffractograms clearly confirms that calcination induces the thermal decomposition of magnesium hydroxide into magnesium oxide according to the dehydration reaction:

7 Mg(OH)_2_ → MgO + H_2_O

During this thermal treatment, the removal of structural water and organic residues leads to lattice reorganization and the formation of highly crystalline MgO nanoparticles without detectable secondary phases.

The crystallite size of the calcined MgO nanoparticles was estimated using the Scherrer equation:8
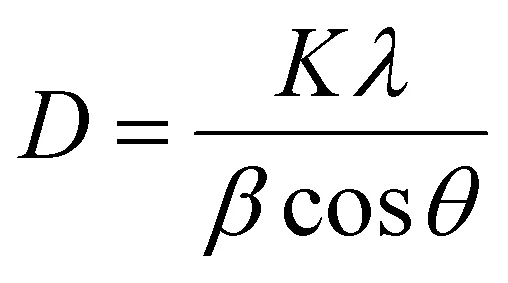
where *D* is the crystallite size, *K* is the shape factor (0.9), *λ* is the X-ray wavelength for Cu Kα radiation (0.15406 nm), *β* is the full width at half maximum (FWHM) of the diffraction peak and *θ* is the Bragg angle.^32,45^

The crystallite size was estimated using the Debye–Scherrer equation based on the most intense diffraction peak, particularly the (200) reflection. The results are summarized in Table 2.

**Table 2:** Crystallite size of MgO nanoparticles calculated from XRD data using the Debye–Scherrer equation

Sample	Peak (2*θ*)	FWHM (*β*)	Cristallite size (nm)
Uncalcined	42.35°	1.078°	7.9
Calcined	42.93°	0.647°	13.2

The calculated crystallite sizes indicate an increase after calcination, attributed to crystal growth and structural reorganization. The nanoscale dimensions are consistent with the observed peak broadening and confirm that the green synthesis route effectively limits crystal growth, which is beneficial for adsorption applications. This result is consistent with previously reported MgO nanoparticles synthesized through similar methods.^43,46^

#### 3.1.2. Fourier transform infra-red (FTIR) spectroscopy

**Fig. 3:**
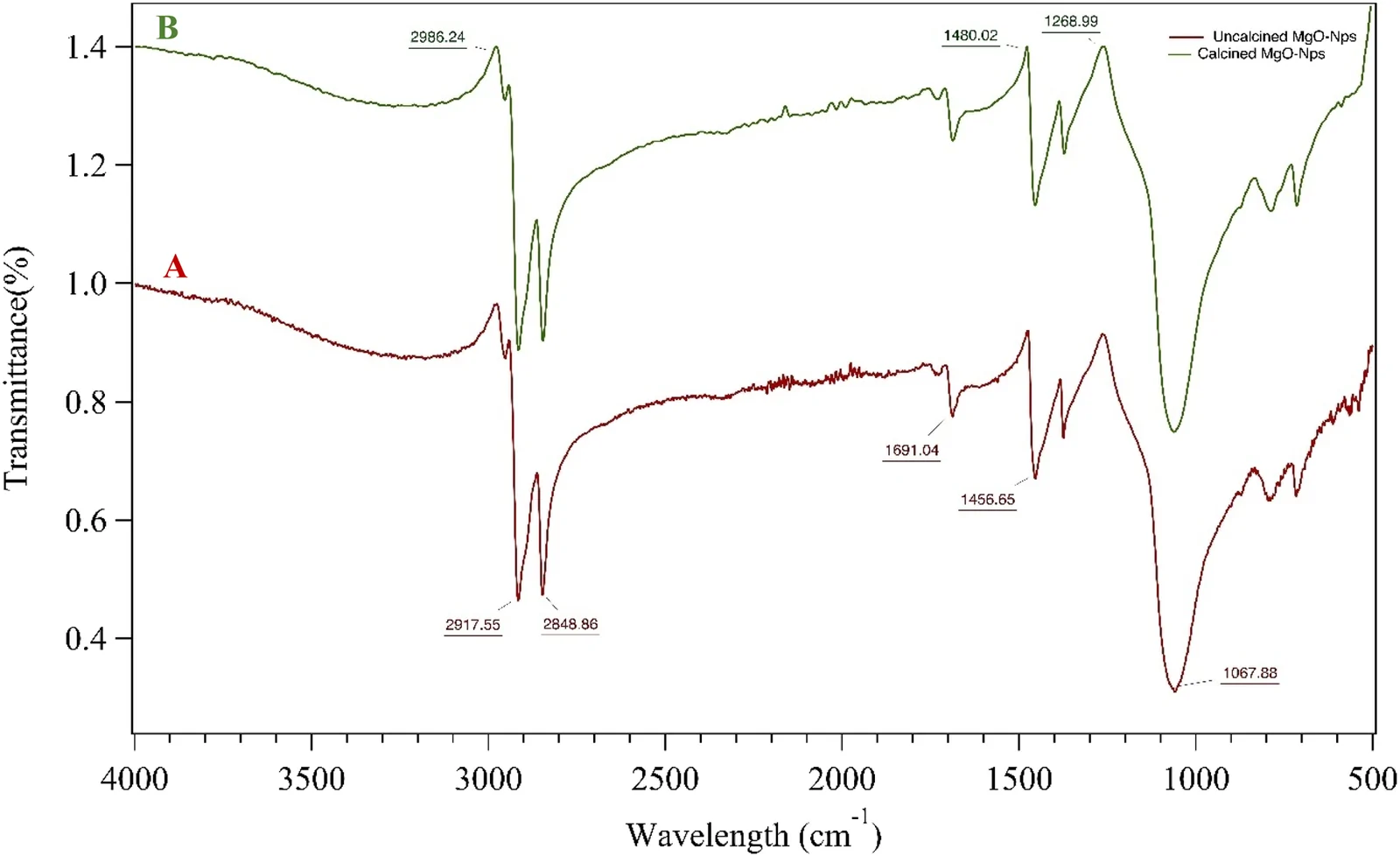
FTIR spectrum of uncalcined MgO NPs (A); calcined MgO NPs (B).

FTIR analysis was performed to identify the surface functional groups of the green-synthesized MgO NPs, as shown in Fig. 3. The spectra were recorded in the wavenumber range of 4000–400 cm^−1^ for both uncalcined and calcined samples.

For the uncalcined sample, the symmetric and asymmetric stretching vibrations of aliphatic C–H bonds in alkane chains are clearly resolved at 2917 and 2848 cm^−1^, which confirms the successful capping of the precursor by phytochemical intermediates from the olive pomace.^47^

The sharp band at 1691 cm^−1^ is assigned to the stretching vibrations of C=O carbonyl group configurations in flavonoids and polyphenolic acids, combined with the bending vibration of interlamellar adsorbed water (H–O–H). Crucially, the strong band at 1456 cm^−1^ reflects the unidentate or bidentate coordination splitting modes of carbonate groups (CO_3_^−2^), resulting from the immediate chemisorption of atmospheric CO_2_ onto the highly basic exposed surface pairs of the nanoparticle precursor. The broad absorption band at 1067 cm^−1^ is conclusively assigned to the C–O–C stretching vibrations of aromatic ethers and glycosidic linkages native to the lignocellulosic matrix of the olive waste.

After calcination, noticeable changes in the FTIR spectrum are observed. The significant reduction in the intensity of bands corresponding to C–H and C=O groups indicate the decomposition and removal of organic residues during thermal treatment.

The band appearing at approximately 1480 cm^−1^ is attributed to carbonate species (CO_3_^2−^), commonly formed due to the interaction of MgO with atmospheric CO_2_.^48,49^

A weak band near 1268 cm^−1^ may be related to residual C–O vibrations, indicating the presence of trace organic species.^50,51^

At lower wavenumbers, the presence of bands below 600 cm^−1^ is attributed to Mg–O lattice vibrations, confirming the formation of magnesium oxide.^47–49^

These results confirm that calcination effectively removes most organic components and promotes the formation of MgO, in agreement with the XRD results. They also indicate that a fraction of surface carbonate species remains adsorbed on the MgO surface, which may play a role in the surface charge and adsorption behavior toward anionic dyes such as Congo Red.

#### 3.1.3. Scanning electron microscopy (SEM) and energy dispersive X-ray spectroscopy (EDX) analysis

**Fig. 4:**
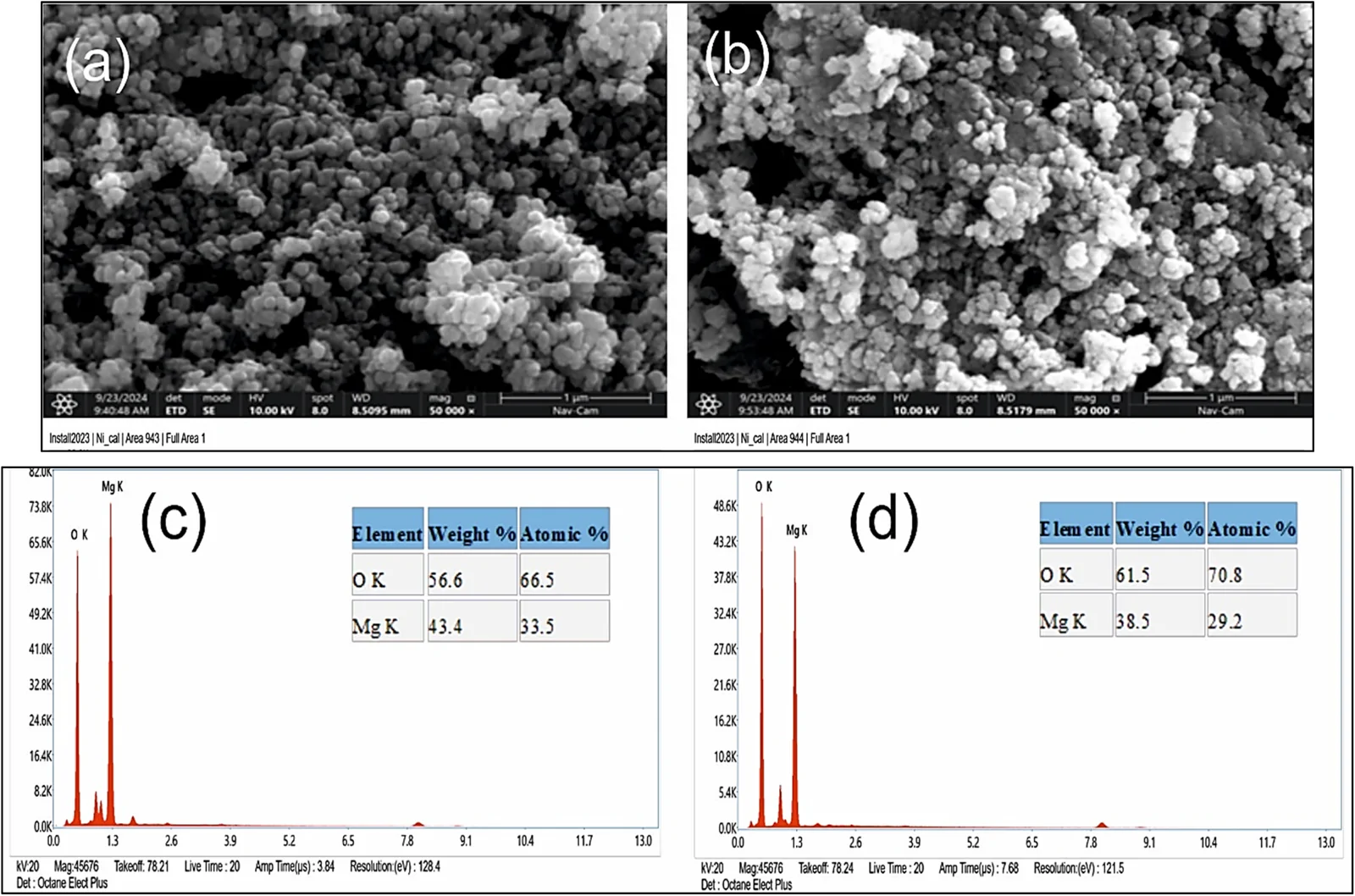
SEM–EDX micrographs: (a–c) calcined MgO NPs and (b–d) uncalcined MgO NPs.

The morphology and particle size distribution of the synthesized MgO NPs were examined using FE-SEM at a magnification of 50.000×, as presented in Fig. 4.

The SEM images reveal a clear difference in morphology before and after calcination. The calcined MgO NPs (Fig. 4a) exhibit relatively more homogeneous and better-defined particles, although some degree of agglomeration is still observed. This behavior can be attributed to the removal of organic residues and the improvement of crystallinity during the calcination process.^52^ In contrast, the uncalcined sample (Fig. 4b) shows highly agglomerated and irregular structures, likely due to the presence of residual phytochemicals and incomplete particle formation.

The particles appear as aggregated clusters composed of finer primary nanoparticles, which is a typical feature of metal oxide nanomaterials due to their high surface energy. The reduction of organic matter after calcination promotes particle reorganization and leads to a more structured morphology. Such morphological evolution may enhance the accessibility of active sites and facilitate mass transfer during adsorption processes; however, adsorption performance also depends on other factors such as surface area and porosity, which are further evaluated by BET analysis.^53,54^

The EDX spectra (Fig. 4c and d) confirm that magnesium and oxygen are the main elements present in both samples, indicating the successful formation of MgO-based materials. For the calcined MgO NPs (Fig. 4c), the weight percentages of Mg and O are 43.4% and 56.6%, respectively, whereas the uncalcined sample (Fig. 4d) shows 38.5% Mg and 61.5% O. This change in composition after calcination suggests the removal of residual organic species and hydroxyl groups, leading to a composition closer to that of stoichiometric MgO. No significant impurity peaks were detected, confirming the high purity of the synthesized material. These findings are consistent with previous studies reporting that calcination improves both the structural organization and chemical purity of MgO nanoparticles.^55,56^ Combined with XRD and FTIR, SEM–EDX thus validates that the green synthesis followed by calcination yields phase-pure MgO with a morphology favorable for adsorption.

#### 3.1.4. Textural characterization of MgO NPs

**Fig. 5:**
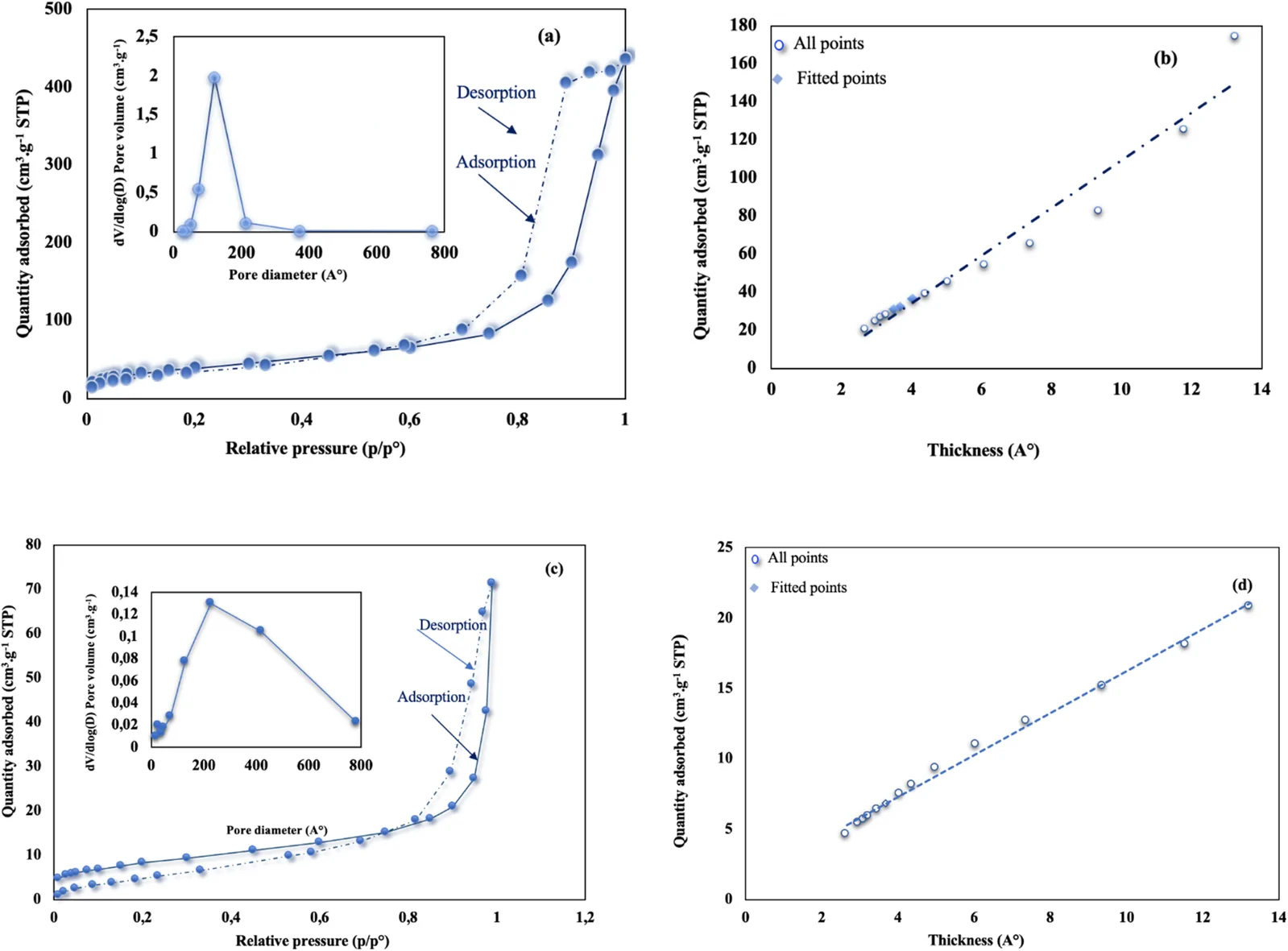
N_2_ adsorption–desorption isotherms at 77 K and corresponding *t*-plots for (a and b) uncalcined MgO NPs and (c and d) calcined MgO NPs.

The nitrogen adsorption–desorption isotherms and corresponding t-plots of MgO NPs at 77 K are presented in Fig. 5. The isotherms exhibit noticeable differences before and after calcination, reflecting significant changes in surface texture and pore structure induced by thermal treatment.

Before calcination, the isotherm can be classified as Type IV with an H3-type hysteresis loop, which is typically associated with porous materials composed of aggregated particles forming slit-shaped pores or interparticle voids.^57^ Such behavior is commonly reported for MgO nanoparticles and other metal oxides, where the porous structure mainly arises from particle aggregation rather than well-defined intrinsic porosity.^57^ The gradual nitrogen uptake at low relative pressure, followed by a sharp increase near *P*/*P*_0_ ≈ 0.9, indicates capillary condensation occurring in mesopores and interparticle voids.^58^ The BET specific surface area was estimated to be 141.96 m^2^ g^−1^.

The BJH pore size distribution reveals an average pore diameter of approximately 23.97 nm, suggesting a mesoporous structure dominated by interparticle voids rather than intrinsic porosity.^57^ It should be noted that the BJH method becomes less accurate for larger pore sizes (>50 nm), and therefore these values should be interpreted with caution.^57^

After calcination, the isotherm retains a Type IV profile with a modified hysteresis loop, indicating structural rearrangement rather than a change in adsorption mechanism. Similar Type IV isotherms have been widely reported for MgO nanostructures synthesized *via* green or precipitation routes.^59^ The BET surface area significantly decreases to 29.40 m^2^ g^−1^. This reduction can be attributed to particle sintering, crystallite growth, and agglomeration induced by thermal treatment. At elevated temperatures, the increased thermal energy enhances atomic diffusion on the surface of MgO nanoparticles, promoting their coalescence into larger structures, leading to a reduction in the specific surface area, as also reported in previous studies.^60,61^

In addition, Calcination leads to particle aggregation, creating larger interparticle pores (∼98.75 nm) and a more compact structure with reduced surface area, as confirmed by XRD and SEM. The porosity mainly comes from spaces between particles rather than intrinsic pores.

This presents an intriguing phenomenon: despite a significant five-fold decrease in the specific BET surface area following calcination (dropping from 141.96 to 29.40 m^2^ g^−1^), the calcined MgO NPs maintain an exceptional Congo Red removal efficiency of over 95%. This explicitly demonstrates that the adsorption process is not strictly governed by physical pore entrapment or intrinsic textural surface area. Instead, it is predominantly driven by surface chemistry and strong electrostatic/chemisorption interactions. While uncalcined samples possess a higher surface area, their active surface sites are extensively masked by amorphous lignocellulosic residues and unreacted organic fractions from the plant extract. Thermal calcination at 600 °C effectively burns off these organic barriers and decomposes the intermediate Mg(OH)_2_ phase, deliberately exposing highly crystalline, unmasked active Mg^2+^ and O^2−^ centers. Consequently, the net density and accessibility of functional chemisorption sites are vastly enhanced after calcination, easily compensating for the loss in raw physical surface area. This explains why the calcined MgO NPs exhibit superior adsorption performance despite their lower BET surface area (Table 3).

**Table 3:** BET and BJH textural parameters of MgO nanoparticles before calcination and after calcination

Parameter	Before calcination	After calcination
BET surface area (m^2^ g^−1^)	141.96	29.40
t-plot external surface area (m^2^ g^−1^)	149.63	30.43
Monolayer capacity, *Q*_m_ (cm^3^ per g STP)	32.61	6.75
BET constant, *C*	80.95	98.40
Total pore volume (cm^3^ g^−1^)	0.673	0.110
BJH adsorption pore volume (cm^3^ g^−1^)	0.660	0.107
BJH desorption pore volume (cm^3^ g^−1^)	0.674	0.108
Average pore diameter (BJH adsorption) (Å)	172.43	162.11
Average pore diameter (BJH desorption) (Å)	109.63	141.80

#### 3.1.5. Thermogravimetric analysis (TG) and derivative thermogravimetry (DTG)

Thermogravimetric analysis (TG) coupled with derivative thermogravimetry (DTG) was employed to investigate the thermal stability and decomposition behavior of the synthesized MgO NPs, as well as the effect of calcination on their surface composition.

**Fig. 6:**
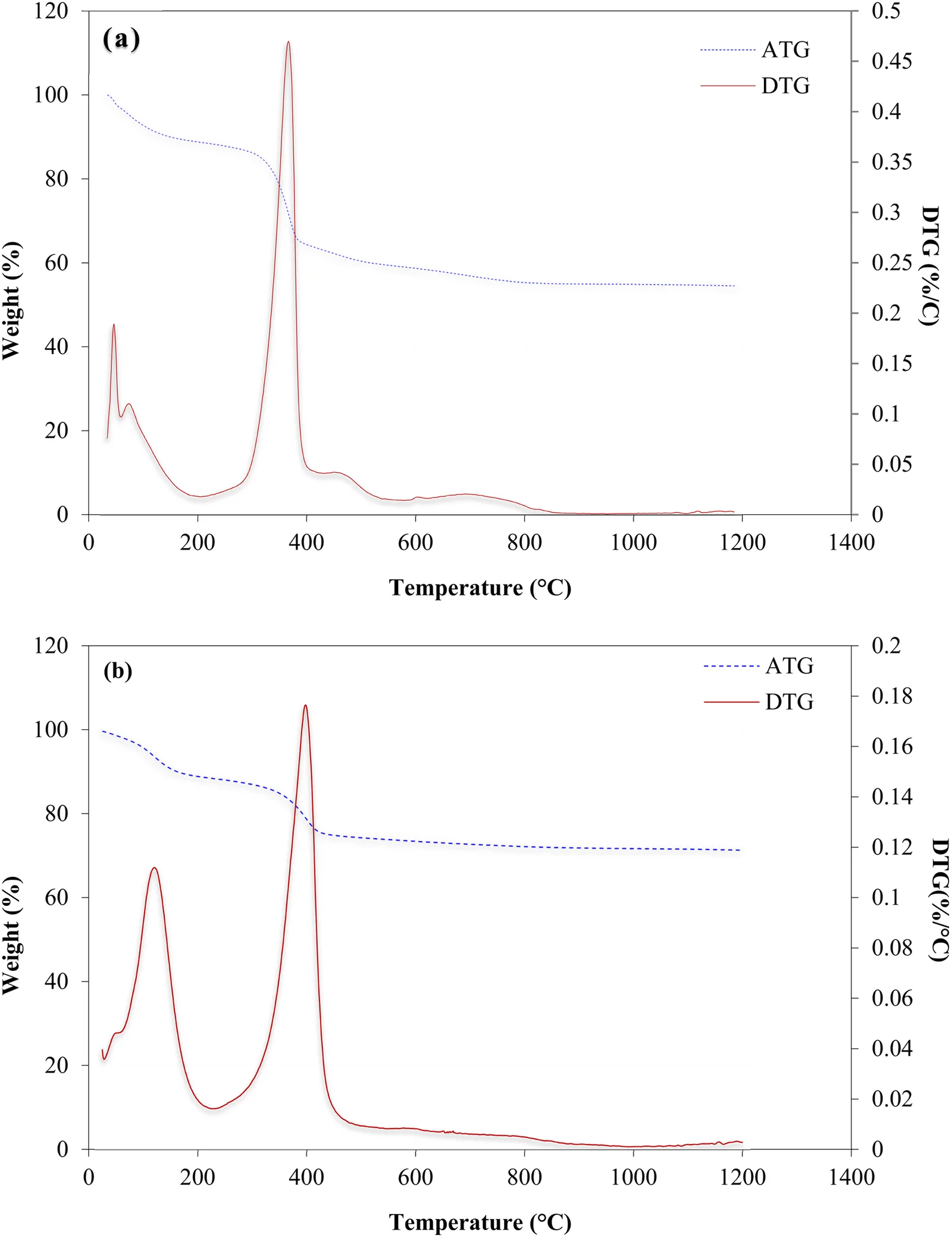
TG–DTG analysis of uncalcined (a) and calcined (b) MgO NPs.

Fig. 6 presents the TG–DTG curves of the uncalcined (a) and calcined (b) MgO NPs, highlighting the influence of thermal treatment on their physicochemical stability and surface characteristics.

For the uncalcined MgO NPs (Fig. 6a), an initial mass loss below ∼200 °C is attributed to the removal of physically adsorbed water and volatile species. A second, more pronounced weight loss observed between ∼300 and 450 °C, accompanied by a strong DTG peak, is attributed to the combined effect of the decomposition of residual organic compounds originating from the plant extract, as well as the dihydroxylation of magnesium hydroxide (Mg(OH)_2_) into MgO according to the reaction:^60^

9 Mg(OH)_2_ → MgO + H_2_O

In addition, part of the weight loss in this temperature range may also be attributed to the release of surface carbonate species (CO_2_) formed by atmospheric adsorption.^60^ These results confirm the presence of hydroxyl groups and organic residues in the uncalcined sample.

In contrast, the calcined MgO NPs (Fig. 6b) exhibit significantly lower weight loss and reduced DTG intensity, indicating the effective removal of organic matter and hydroxyl species during calcination. The negligible mass variation above 500 °C demonstrates the high thermal stability of the calcined MgO, which is consistent with the formation of a well-crystallized MgO phase as confirmed by XRD analysis.

The improved thermal stability after calcination can be attributed to increased crystallinity and particle growth, which reduce the number of reactive surface sites and enhance structural stability. Similar trends have been reported in previous studies, where increasing calcination temperature leads to improved thermal resistance due to crystallite growth and structural densification.^50,60^

Overall, these results confirm that calcination plays a crucial role in improving the thermal stability and purity of MgO nanoparticles while modifying their surface chemistry. This thermal behavior is consistent with the removal of lignocellulosic residues and the transformation of Mg(OH)_2_ into MgO, as inferred from XRD and FTIR analyses.

### 3.2. Study of CR dye adsorption

#### 3.2.1. Effect of adsorbent mass

The adsorbent mass is a key parameter influencing the adsorption performance, as it directly affects the number of available active sites and the surface area accessible for pollutant removal.^62^

The effect of MgO NPs mass on the adsorption of Congo Red (CR) was investigated by varying the adsorbent mass from 0.1 to 0.6 g under constant experimental conditions (pH = 7.36, temperature = 25 °C, initial dye concentration = 20 mg L^−1^, and contact time = 180 min).

**Fig. 7:**
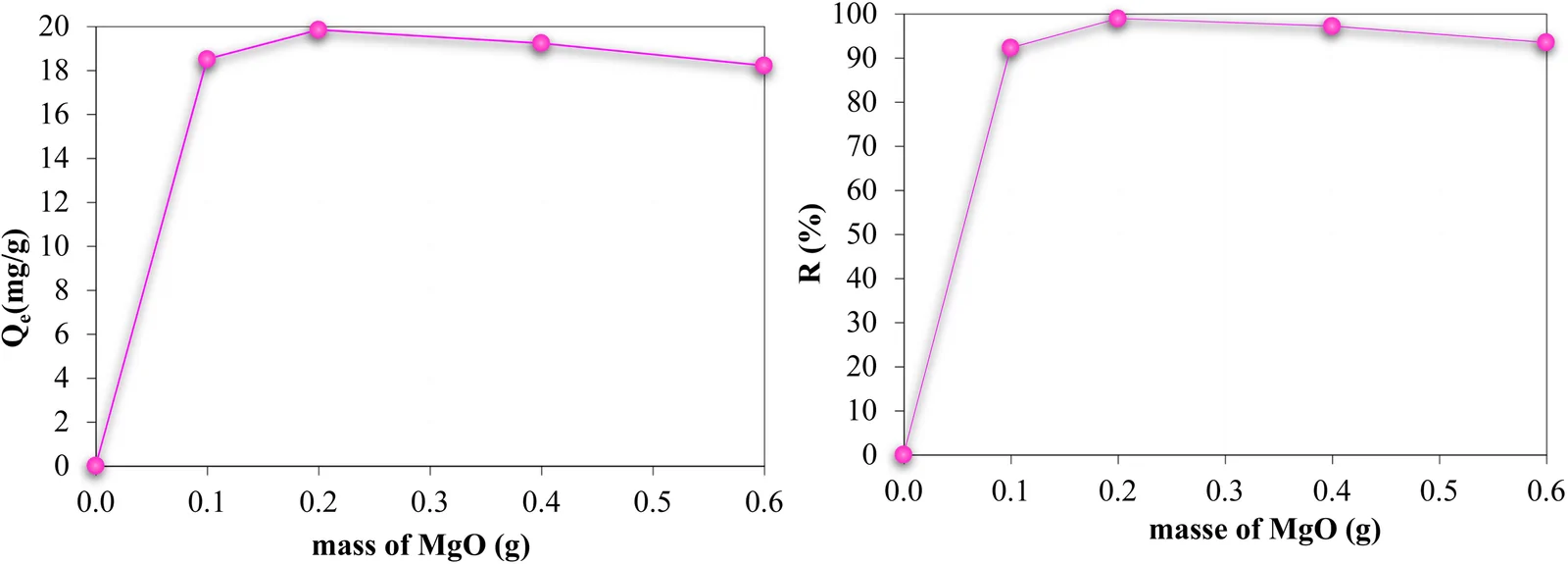
Effect of MgO NPs dosage on adsorption performance: (a) equilibrium adsorption capacity (*q*_e_) and (b) removal efficiency of Congo Red. ([CR] = 20 mg L^−1^, pH ≈ 7 and *T* = 25 °C).

As shown in Fig. 7, the adsorption capacity increases from 18.5 mg g^−1^ (92.27%) at 0.1 g to a maximum value of 19.83 mg g^−1^ (98.91%) at 0.2 g, corresponding to a removal efficiency of approximately 98.9%. This initial improvement can be attributed to the higher availability of active sites and the increase in accessible surface area, which enhances the interaction between Congo Red molecules and the adsorbent surface.^63,64^

Beyond this dosage, only slight variations in adsorption capacity are observed, with values of 19.5 mg g^−1^ at 0.4 g and 18.75 mg g^−1^ at 0.6 g. This behavior indicates that the adsorption process has reached a saturation state due to the fixed initial dye concentration.^63,65,66^

At higher adsorbent dosages, the number of available active sites exceeds the amount of dye molecules, leading to inefficient utilization of the adsorbent and a slight reduction in adsorption capacity per unit mass. In addition, particle aggregation at higher dosages may reduce the effective surface area and limit the accessibility of some active sites.^63,65,66^

Therefore, 0.2 g was identified as the optimal adsorbent dosage, as it provides the highest adsorption capacity while ensuring efficient use of the adsorbent material. This mass was consequently selected for subsequent kinetic, isotherm, and thermodynamic studies.

**Fig. 8:**
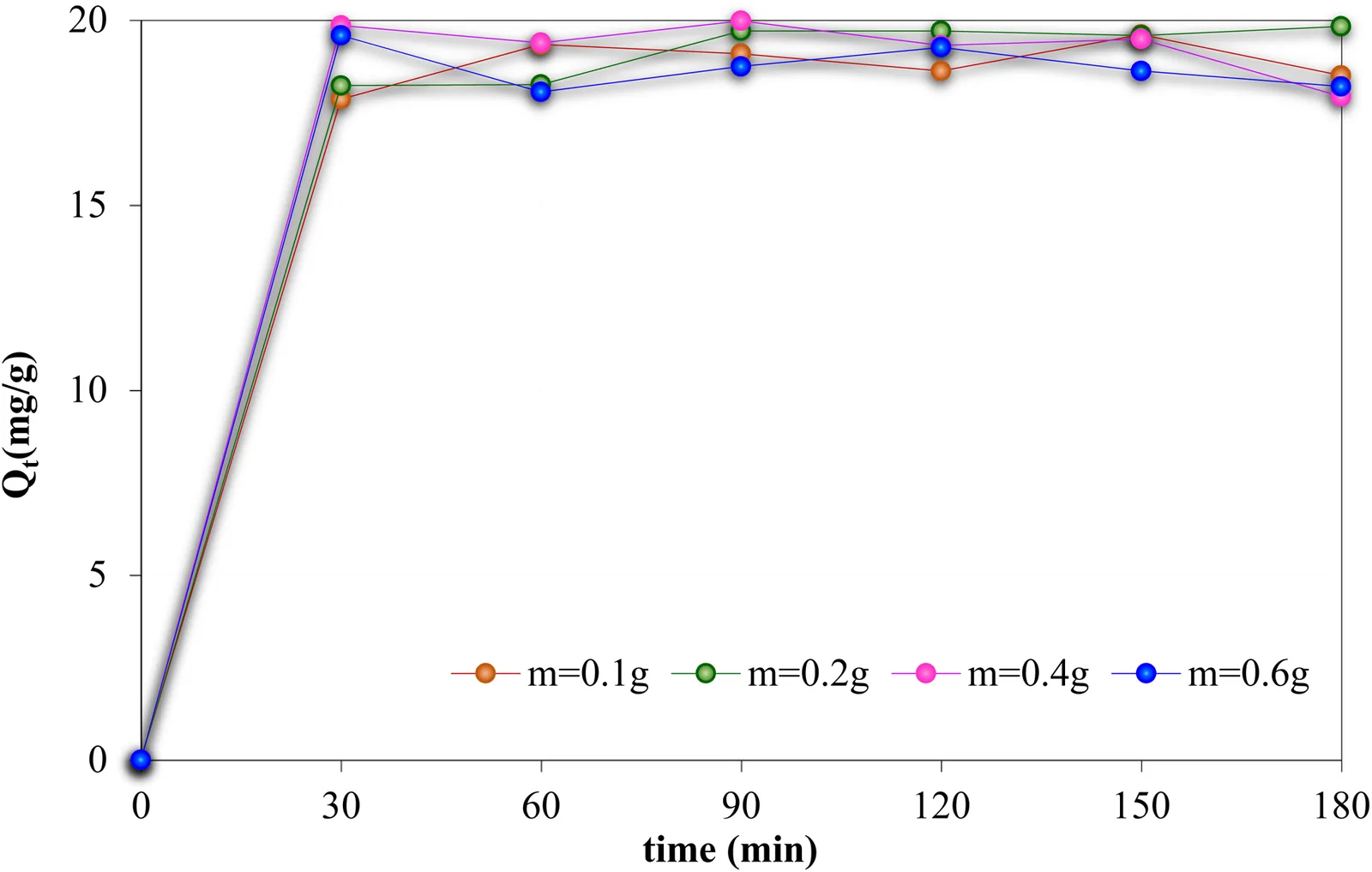
Effect of contact time on the adsorption capacity of Congo Red onto MgO NPs at different adsorbent dosages ([CR] = 20 mg L^−1^, pH ≈ 7 and *T* = 25 °C).

As shown in Fig. 8, the adsorption process is characterized by a rapid initial uptake, followed by a slower approach to equilibrium. Approximately 92% of CR is removed within the first 30 minutes, indicating a fast adsorption rate. This initial stage can be attributed to the abundance of readily available active sites on the surface of MgO NPs, which facilitates strong interactions between the dye molecules and the adsorbent.^67,68^

As the contact time increases, the adsorption rate gradually decreases, and the system reaches equilibrium at around 90 minutes. This slowdown is mainly due to the progressive occupation of active sites and the decrease in the concentration gradient between the solution and the adsorbent surface, which reduces the driving force of mass transfer.^65,69,70^ Beyond 90 minutes, no significant improvement in adsorption is observed, confirming that equilibrium has been attained.^66^ Therefore, 90 minutes was selected as the optimal contact time for subsequent experiments.

These results suggest that the adsorption of CR onto MgO NPs involves a rapid surface interaction followed by a slower diffusion-controlled stage, which is commonly observed in adsorption processes involving porous nanomaterials. This behavior is later corroborated by kinetic modeling, which reveals that the pseudo-second-order model best describes the experimental data, consistent with a chemisorption-controlled mechanism.

#### 3.2.2. Effect of Congo red concentration

The effect of initial CR concentration on the adsorption performance of MgO nanoparticles was investigated by varying the dye concentration from 20 to 80 mg L^−1^, while keeping all other experimental parameters constant.

**Fig. 9:**
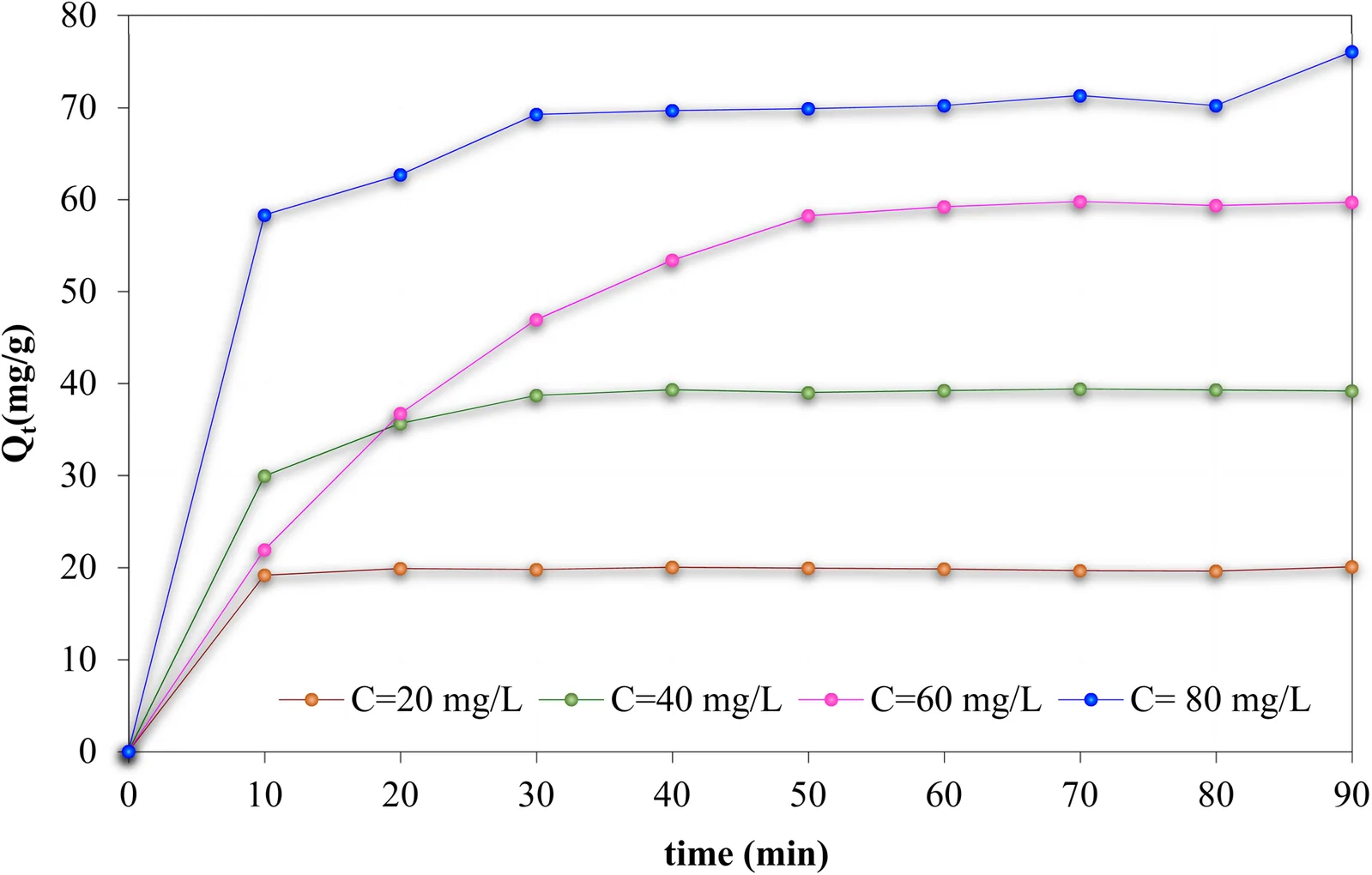
Effect of initial dye concentration on Congo Red adsorption (pH ≈ 7, *T* = 25 °C and *m*_MgO_ = 0.2 g).

As illustrated in Fig. 9, increasing the initial CR concentration from 20 to 80 mg L^−1^ led to an increase in adsorption capacity from 20.38 to 76.1 mg g^−1^. This behavior can be attributed to the enhancement of the mass transfer driving force at higher concentrations, which facilitates the diffusion of CR molecules toward the active sites of the adsorbent.^71,72^

Regarding the removal efficiency, high values were observed at lower concentrations (around 98%), followed by a noticeable decrease at higher concentration (94.01% at 80 mg L^−1^). This behavior can be attributed to the progressive saturation of active sites on the MgO nanoparticle surface, which limits the availability of adsorption sites at higher dye concentrations.^73^ Thus, at elevated CR loadings, the system approaches monolayer coverage and the adsorption capacity increases at the expense of removal percentage.

#### 3.2.3. Effect of ionic strength

Several ions, including cations (K^+^, Na^+^, Ca^2+^) and anions (NO_3_^−^, Cl^−^, H_2_PO_4_^−^, SO_4_^2−^, CO_3_^2−^), are commonly present in wastewater and natural waters. Their presence can significantly influence the adsorption of CR by competing for active sites and altering the surface charge of the adsorbent, which justifies the investigation of the effect of salt.^74^ The effect of ionic strength on the adsorption of CR onto MgO nanoparticles was evaluated by adding different concentrations of KCl under constant experimental conditions.

**Fig. 10:**
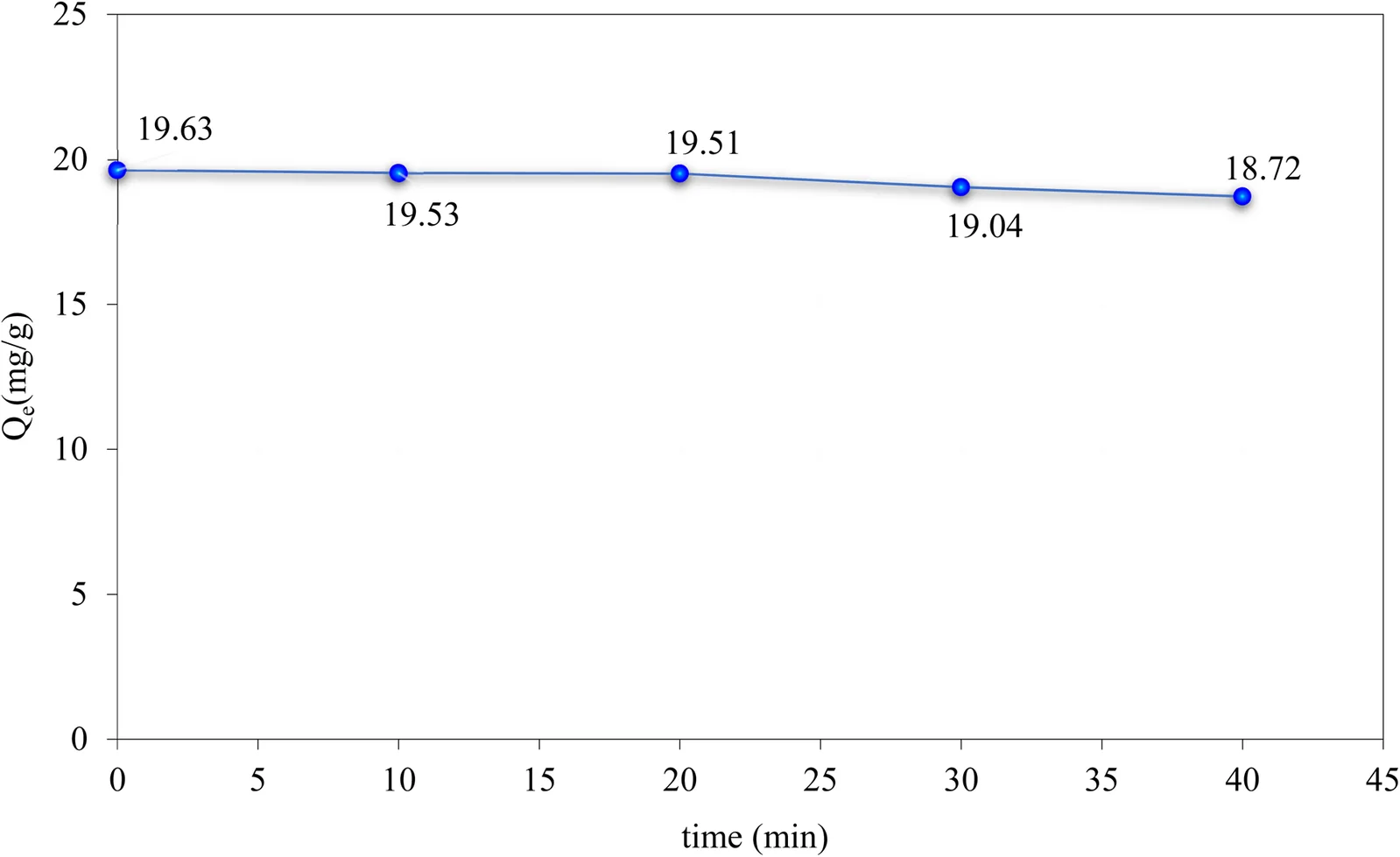
Effect of KCl concentration on adsorption of CR dye ([CR] = 20 mg L^−1^, pH ≈ 7, *T* = 25 °C and *m*_MgO_ = 0.2 g).

As shown in Fig. 10, the equilibrium adsorption capacity (*q*_e_) slightly decreased from 19.63 to 19.51 mg g^−1^ at low KCl concentrations (0–20 mg L^−1^), indicating that the presence of a small amount of ions does not significantly hinder the adsorption process.^75^

However, at higher KCl concentrations, *q*_e_ gradually decreased to 18.72 mg g^−1^. This decline can be attributed to the screening effect, where K^+^ and Cl^−^ ions compete with CR molecules for the available active sites and shield the surface charge of MgO nanoparticles, thereby reducing the electrostatic attraction between the adsorbent and the dye molecules.^76,77^

These results suggest that, although MgO NPs remain efficient in the presence of moderate ionic strength, high salt contents typical of saline industrial effluents may partially inhibit CR adsorption and should be considered when designing real-scale applications.

#### 3.2.4. Effect of pH

**Fig. 11:**
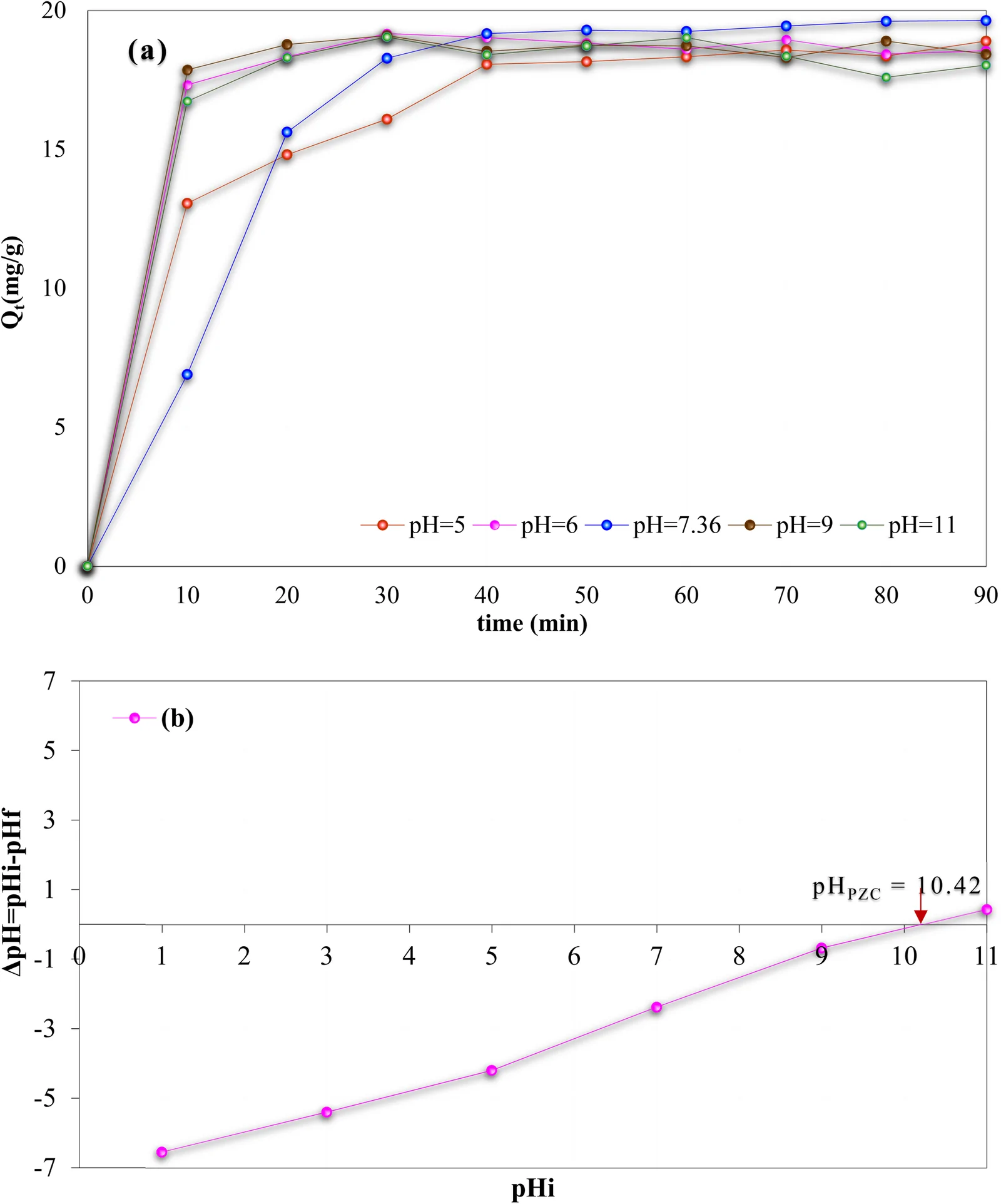
(a) Effect of pH on adsorption of CR ([CR] = 20 mg L^−1^, initial pH = 7.36, *T* = 25 °C, *m*_MgO_ = 0.2 g), (b) pH_PZC_ of MgO NPs.

The initial pH of the dye solution plays a crucial role in the adsorption process, as it governs both the surface charge of the adsorbent (MgO NPs) and the ionization state of the adsorbate (Congo Red, CR).^78^ As illustrated in Fig. 11, the adsorption capacity is dependent on the solution pH.

Experiments performed at an initial CR concentration of 20 mg L^−1^ and an adsorbent dose of 0.2 g L^−1^ showed that the maximum adsorption capacity (19.63 mg g^−1^) was achieved at pH 7.36, which represented the natural pH of the solution.

When the pH is lower than the pH_PZC_ (10.42), the surface of MgO NPs is positively charged due to protonation (formation of Mg–OH_2_^+^ species). Since Congo Red is an anionic dye, strong electrostatic attraction occurs between the negatively charged dye molecules and the positively charged adsorbent surface, resulting in enhanced adsorption. Similar behavior has been reported in the literature, where adsorption increases with increasing pH in the acidic range due to reduced proton competition and improved electrostatic interactions.^57^

In slightly acidic conditions (pH 5–6), a marginal decrease in adsorption capacity (18.9–18.55 mg g^−1^) was observed. This can be attributed to the competition between excess H^+^ ions and dye molecules for active adsorption sites, as well as possible changes in dye aggregation behavior.

At near-neutral pH (≈7), optimal adsorption is observed, which can be explained by a balance between electrostatic attraction and reduced competition effects. This observation is consistent with previous studies reporting maximum CR adsorption around neutral pH for MgO-based adsorbents.^57,67^

Conversely, at alkaline pH values (9–11), the adsorption capacity slightly decreases (18.42–18.03 mg g^−1^). As the pH approaches or exceeds the pH_PZC_, the MgO surface becomes less positively charged and eventually negatively charged due to the formation of deprotonated species (Mg–O^−^). This leads to electrostatic repulsion between the negatively charged surface and anionic CR molecules, thereby reducing adsorption efficiency. Similar trends have been reported for MgO-based nanoadsorbents, where adsorption decreases at higher pH due to surface charge reversal and electrostatic repulsion.^64^

Overall, the results confirm that electrostatic interactions are the dominant mechanism governing CR adsorption onto MgO NPs. The optimal adsorption observed near neutral pH reflects a favorable balance between surface charge and dye speciation, in agreement with previous studies on similar systems.^57,67^ This finding is fully consistent with the electronic structure analysis of CR, which shows highly nucleophilic sulfonate groups interacting most efficiently with protonated Mg–OH_2_^+^ sites around neutral pH.

### 3.3. Study of adsorption isotherm

The equilibrium adsorption behavior of Congo Red (CR) onto MgO nanoparticles (MgO NPs) was investigated at 293, 303, and 313 K. The adsorption process was evaluated using non-linear forms of four widely applied isotherm models, namely Langmuir, Freundlich, Temkin, and Dubinin–Radushkevich (D–R).

The Langmuir model^55,79–81^ assumes monolayer adsorption onto a homogeneous surface with identical active sites, whereas the Freundlich model describes adsorption on heterogeneous surfaces.^55,62,82,83^ The Temkin model considers adsorbate–adsorbent interactions,^84,85^ while the D–R model provides insight into the adsorption energy and mechanism.^86–88^

**Fig. 12:**
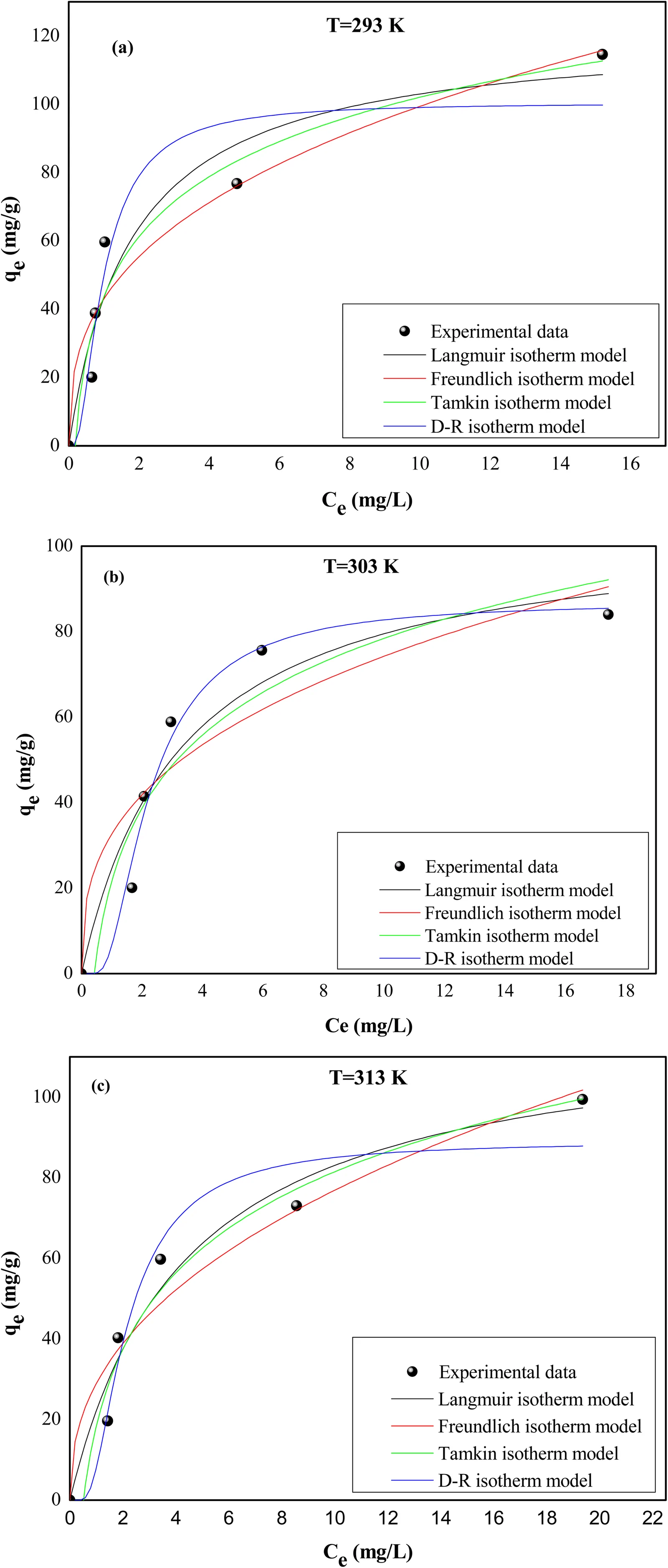
Nonlinear isotherms plot for CR adsorption onto MgO NPs, (a): *T* = 293 K, (b): 303 K and (c): 313 K.

The mathematical expressions of these models and their corresponding parameters are summarized in Table 4, while the nonlinear fitting curves are presented in Fig. 12.

**Table 4:** Isotherm models, equations, and fitted parameters for CR adsorption onto MgO NPs

Model	Equation	Parameter	Unit	293 K	303 K	313 K
Langmuir	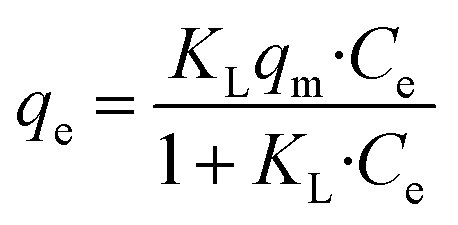 (10)	*q*_m_	mg g^−1^	121.601	105.752	98.737
		*K*_L_	L mg^−1^	0.554	0.303	0.273
		*R* ^2^	—	0.932	0.924	0.903
		SSE	—	575.680	404.408	214.249
Freundlich	*q*_e_ = *K*_F_*C*^1/*n*^_e_ (11)	*K*_F_	(mg g^−1^)(L mg^−1^)^(1/n)	43.006	32.640	28.739
		*n*	—	2.749	2.803	2.645
		*R* ^2^	—	0.934	0.864	0.862
		SSE	—	551.912	402.975	642.410
		RMSE	—	11.746	13.434	12.673
Temkin	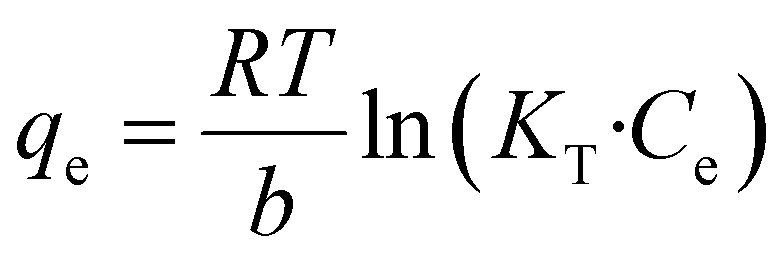 (12)	*K*_T_	L mg^−1^	25.394	24.681	27.384
		*B*	J mol^−1^	5.563	2.397	1.960
		*R* ^2^	—	0.946	0.909	0.894
		SSE	—	470.131	484.709	495.352
		RMSE	—	10.841	11.008	11.129
D–R	*q*_e_ = *q*_max_exp(−*Bε*^2^) (13)	*q*_m_	mg g^−1^	100.394	86.890	80.047
		*B*	mol^2^/kJ^2^	1.460	5.395	6.322
		*E*	kJ mol^−1^	8.89	9.63	18.55
	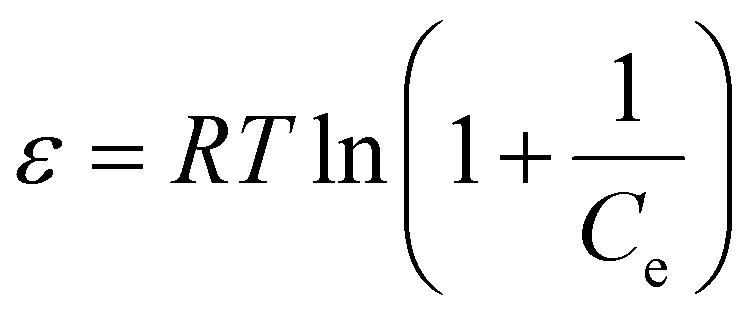 (14)	*R* ^2^	—	0.920	0.986	0.954
		SSE	—	753.233	75.894	211.914
		RMSE	—	13.722	4.355	7.279

The fitting quality of the investigated isotherm models was evaluated using multiple statistical criteria, including the correlation coefficient (*R*^2^), sum of squared errors (SSE), and root mean square error (RMSE), to ensure a reliable and unbiased comparison of the models.^89^

At 293 K, the Temkin model provided the best overall fit (*R*^2^ = 0.946, SSE = 470.131, RMSE = 10.841), indicating the importance of adsorbate–adsorbent interactions at lower temperatures. At 303 K and 313 K, the D–R model exhibited the best fitting performance, with the highest *R*^2^ values (0.986 and 0.954) and lowest SSE/RMSE (75.894/4.355 and 211.914/7.279), confirming that adsorption at higher temperatures is strongly governed by the energetic heterogeneity of the MgO surface, consistent with the Gaussian energy distribution assumed in the D–R model.^90^

The maximum adsorption capacities (*q*_m_) from the Langmuir model decreased from 121.601 to 98.737 mg g^−1^ with increasing temperature, reflecting the exothermic nature of the adsorption process. Similarly, the D–R model predicted *q*_m_ values ranging from 100.394 to 80.047 mg g^−1^, which corroborates the temperature-dependent adsorption trend.^64^ It is important to note that these *q*_m_ values reflect adsorption under liquid-phase conditions and cannot be directly compared to the BET specific surface area, which measures nitrogen adsorption at 77 K.

The Freundlich model yielded *n* values greater than unity at all temperatures, indicating favorable adsorption on a heterogeneous surface.^62^ Nevertheless, its lower *R*^2^ values and higher error functions (RMSE and SSE) compared to the Temkin and D–R models demonstrate that it does not provide the most accurate representation of the adsorption equilibrium.

Further insight into the adsorption mechanism was obtained from the D–R model through the mean adsorption energy (E), which ranged from 8.89 to 18.55 kJ mol^−1^, indicating that the adsorption mechanism involves a combination of physical and chemical interactions. Values of *E* between 8 and 16 kJ mol^−1^ suggest predominantly ion-exchange or physisorption interactions, whereas values above 16 kJ mol^−1^ at higher temperature indicate a shift toward chemisorption.^67,70,91^

Overall, the combined analysis of all isotherm parameters, model errors, and adsorption energies demonstrates that CR adsorption onto MgO NPs occurs on a heterogeneous surface with a mixed mechanism, evolving from interaction-controlled (Temkin-dominated) behavior at lower temperatures to energy-controlled (D–R-dominated) adsorption at higher temperatures, confirming the presence of both physisorption and chemisorption contributions. This mixed mechanism is in line with the kinetic and thermodynamic results, which point to fast surface interactions combined with deeper adsorption sites accessible through diffusion.

Table 5 presents a comparison of the maximum adsorption capacities (*q*_max_) of various MgO-based and related nanomaterials for dye removal reported in the literature. It is evident that adsorption performance strongly depends on the synthesis method, morphology, and experimental conditions. Advanced engineered MgO nanostructures, such as solid-state synthesized MgO, exhibit very high adsorption capacities (2375 mg g^−1^),^53^ whereas simpler or more sustainable green synthesis methods, including the MgO nanoparticles prepared in this work, achieve moderate but meaningful adsorption capacities (114.6 mg g^−1^ for Congo Red). Despite being lower than some highly engineered adsorbents, our green-synthesized MgO NPs offer the advantages of low-cost, eco-friendly production and ease of preparation, making them suitable for practical wastewater treatment. The large variations in *q*_max_ among different studies highlight the influence of particle size, surface area, porosity, and dye type on adsorption efficiency.

**Table 5:** Comparison of adsorption capacity of different adsorbents for Congo Red removal reported in the literature

Adsorbent	Dye	*q*_max_ (mg g^−1^)	Reference
MgO nanoparticles (solid-state synthesis)	Congo red	2375	53
SnO_2_–TiO_2_ composite	Congo red	39.1	40
Nano-MgO (molten salt method)	Congo red	1100	67
MgO nanoparticles (microwave-assisted synthesis)	Congo red	136	84
MgO nanoparticles (auto-combustion synthesis)	Congo red	14	64
Mesoporous MgO nanorods (sol–gel, CTAB)	Fast orange 3R/Bromophenol blue	30/40	62
MgO NPs (this work)	Congo red	114.6	This study

A comparison of waste-based green synthesis approaches clearly shows that the olive pomace route stands out, offering a unique combination of structural support and functional properties compared to other methods reported in the literature. While conventional fruit waste extracts such as orange peels (Munjal *et al.*),^92^ pomegranate peels (Fouda *et al.*),^93^ lemon peels (Tamam *et al.*),^1^ grape pomace (Edwin *et al.*)^94^*yield* varying dimensions, surface properties, and primary biological or photocatalytic target behaviors, our olive pomace extract offers a naturally rich matrix, combining lignocellulosic structure and polyphenolic compounds, making it an excellent template for synthesis.^95^ This native chemical matrix suppresses severe crystal growth during calcination, yielding finely sized crystalline domains (∼13.2 nm) with highly unmasked active Mg^2+^ chemisorption centers. This directly drives a competitive liquid-phase Congo Red adsorption capacity of 114.6 mg g^−1^ under ambient conditions while simultaneously fulfilling local circular bioeconomy waste-repurposing parameters. (Rev1C1).

It is critical to distinguish between the experimentally achieved operational capacity (*q*_e_ = 19.83 mg g^−1^), which reflects a low initial dye loading condition (*C*_0_ = 20 mg L^−1^) designed to simulate typical industrial effluent baseline exposures, and the modeled maximum adsorption capacity parameter (*q*_max_ = 114.6 mg g^−1^) derived mathematically from the Langmuir isotherm at high saturation limits. The experimental loading of 19.83 mg g^−1^ indicates that under operational conditions, nearly 99% of the dye is successfully captured, leaving the vast majority of the calculated total capacity (114.6 mg g^−1^) available for significantly higher structural pollutant shocks.

### 3.4. Kinetic study

Kinetic studies are essential for understanding the adsorption mechanism, as they provide insights into the rate of pollutant removal and the nature of the interactions between adsorbate molecules and the adsorbent surface.^96,97^ In the present work, the adsorption kinetics of Congo red (CR) onto MgO NPs were evaluated using three commonly applied models: pseudo-first-order (PFO), pseudo-second-order (PSO), and intra-particle diffusion (IPD). These models describe the external mass transfer, surface adsorption, and diffusion mechanisms involved in the overall adsorption process.

The pseudo-first-order (PFO) model (Lagergren model) assumes that the adsorption rate is proportional to the difference between the equilibrium adsorption capacity and the amount of adsorbate adsorbed at time *t*.^98^ The nonlinear form of the model is expressed as:^99–101^

15 ln(*q*_e_ − *q*_*t*_) = ln *q*_e_ − *K*_1_*t*

where *q*_*t*_ (mg g^−1^) is the adsorption capacity at time *t*, *q*_e_ (mg g^−1^) is the equilibrium adsorption capacity, and K_1_ (1/min) is the rate constant of the pseudo-first-order model.

The pseudo-second-order (PSO) model, proposed by Ho and McKay, assumes that chemisorption governs the adsorption process and that the rate-limiting step involves valence forces through sharing or exchange of electrons.^99,102^ The nonlinear expression of the PSO model is given by:^99,102^16
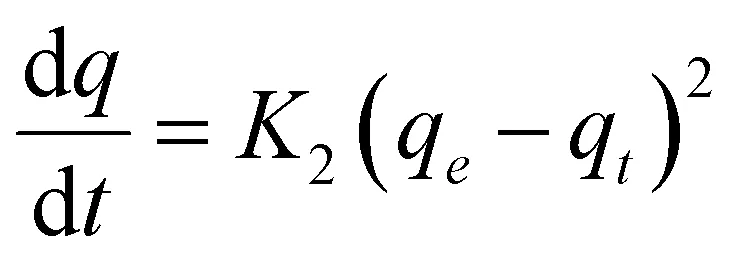
where *K*_2_ (g mg^−1^ min^−1^) is the rate constant of the pseudo-second-order model.

To further investigate the diffusion mechanism, the intra-particle diffusion (IPD) model proposed by Weber and Morris was applied.^103^ This model describes the diffusion of adsorbate molecules within the pores of the adsorbent and is expressed as:^103^

17 *q*_*t*_ = *K*_i_*t*^0.5^ + *C*

**Fig. 13:**
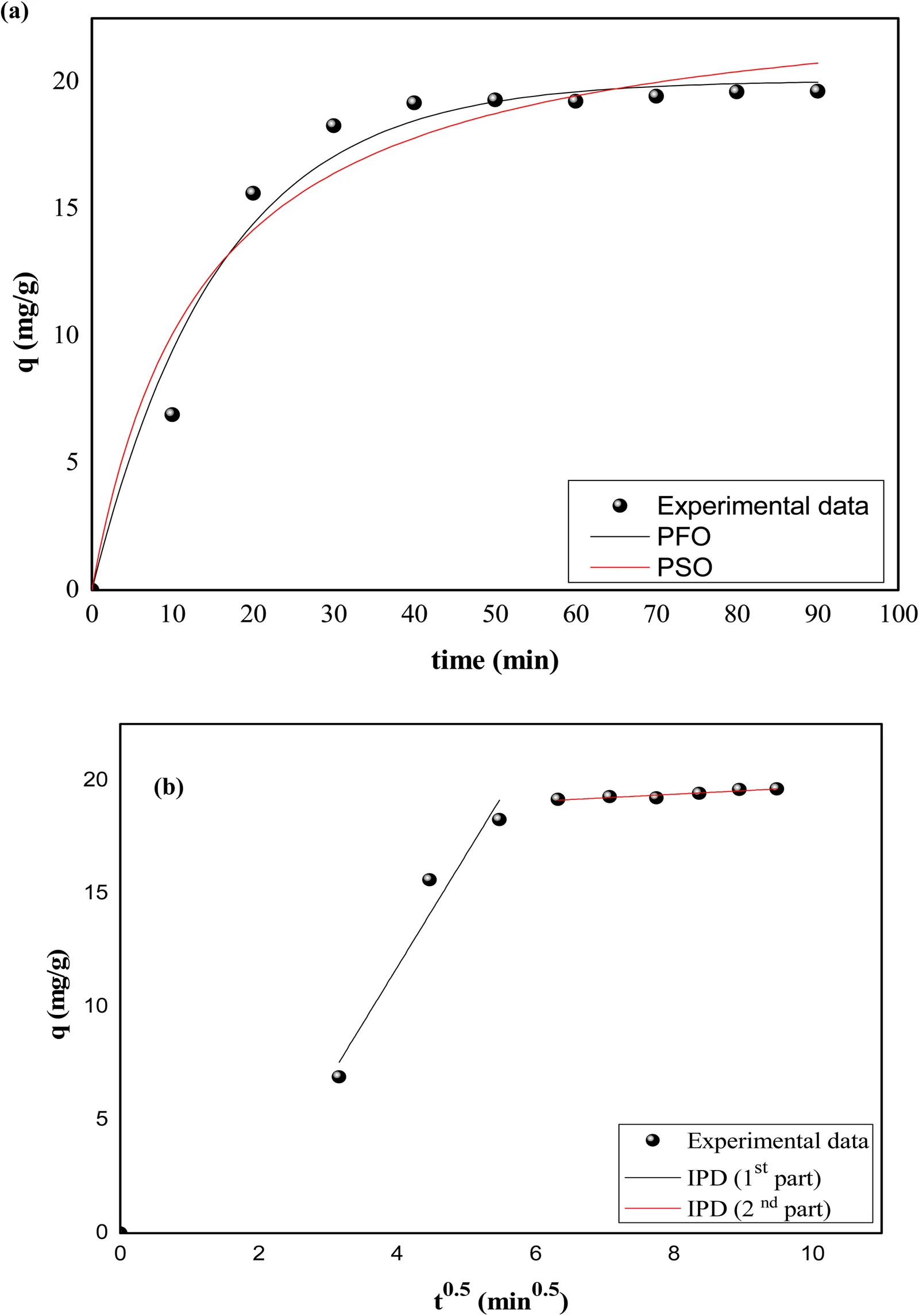
(a) Nonlinear fitting of kinetic models for CR adsorption; (b) intraparticle diffusion (IPD) plot for CR adsorption.

The fitted curves and kinetic model parameters obtained from nonlinear fitting are presented in Fig. 13 and Table 6, respectively.

**Table 6:** Kinetic modeling parameters for CR adsorption onto MgO NPs

Model	Parameter	Value	AIC value	BIC value
Pseudo-first-order (PFO)	*q*_eexp_ (mg g^−1^)	24.453		
	*q*_ecalc_ (calculated) (mg g^−1^)	20.052	45.32	46.12
	*K*_1_ (1/min)	0.063		
	*R* ^2^	0.975		
Pseudo-second-order (PSO)	*q*_eexp_ (mg g^−1^)	24.453		
	*q*_ecalc_ (calculated) (mg g^−1^)	23.963	12.45	13.25
	*K*_2_ (g (mg^−1^ min^−1^)	0.003		
	*R* ^2^	0.951		
Intraparticle diffusion (IPD)	Stage 1 *K*i_1_ (mg/(g min^1^/^2^))	2.145		
	*C*_1_ (mg g^−1^)	6.656	_	_
	*R*_1_^2^	0.989		
	Stage 2 *K*_i2_ (mg/(g min^1^/^2^))	12.542		
	*C*_2_ (mg g^−1^)	1.047		
	*R*_2_^2^	0.974		

To provide an unbiased validation of the rate-controlling step without relying solely on standard correlation parameters (*R*^2^), a comprehensive statistical evaluation was performed using Akaike's Information Criterion (AIC) and Bayesian Information Criterion (BIC). At an ambient baseline temperature of 293 K, the PFO model exhibits a superficially higher correlation coefficient (*R*^2^ = 0.975) compared to the PSO model (*R*^2^ = 0.951). However, the PFO model exhibits a severe deviation of nearly 18% between its calculated equilibrium capacity (20.052 mg g^−1^) and the true experimental boundary (24.453 mg g^−1^). When evaluated through information criteria, the PSO model yields significantly lower values (AIC = 12.45; BIC = 13.25) than the PFO framework (AIC = 45.32; BIC = 46.12). This robust statistical difference conclusively rejects the PFO pathway, demonstrating that the higher *R*^2^ parameter was an artifact of localized experimental scatter rather than true mechanistic modeling. The minimal AIC/BIC values verify that the PSO framework provides a more realistic description of the system, confirming that chemical rate-limiting valence forces through electron sharing or charge transfer govern the adsorption process. These findings are consistent with the strong electrostatic and charge-transfer interactions inferred from the DFT analysis of Congo Red and MgO surface sites.

The IPD model revealed a multi-linear behavior with two distinct stages, indicating that the adsorption process occurs in multiple steps. The first stage corresponds to rapid external surface adsorption (film diffusion), while the second stage is attributed to a slower intra-particle diffusion process occurring within the pores of the adsorbent.^104^

The non-zero intercept (*C* ≠ 0) suggests that intra-particle diffusion is not the sole rate-limiting step and that boundary layer effects also play a significant role in the adsorption mechanism.^62^

Overall, the adsorption process follows a complex multi-step mechanism, where surface interactions and diffusion phenomena play a dominant role, with possible contributions from chemisorption. This multi-step nature matches well with the isotherm and MD results, which show both strong surface binding and progressive occupation of deeper sites within the aggregated MgO structure.

### 3.5. Thermodynamic study

The thermodynamic study of adsorption is essential for understanding the spontaneity, heat changes, and disorder associated with the adsorption process through the evaluation of key parameters, namely Gibbs free energy (Δ*G*°), enthalpy (Δ*H*°), and entropy (Δ*S*°). The thermodynamic behavior of CR adsorption onto MgO NPs was investigated at three different temperatures.^99^ The thermodynamic parameters were calculated using the following equations:^105–107^

18 Δ*G*^°^ = −*RT* ln *K*_c_

19 Δ*G*^°^ = Δ*H*^°^ − *T*ΔS^°^

20
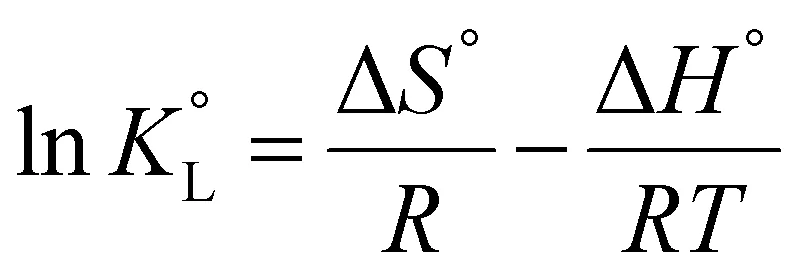
where *R* (8.314 J (mol^−1^ K^−1^)) is the universal gas constant and *T* the temperature in (K). The dimensionless Langmuir equilibrium constant 
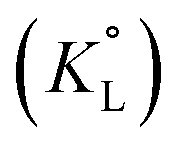
 was derived from the Langmuir constant (*K*_l_, L mg^−1^), obtained from the Langmuir isotherm model (eqn (8)) and reported in Table 4. For thermodynamic calculations, *K*_L_ was converted into a dimensionless form by transforming it into a molar concentration basis, considering the standard state (*C*_0_ = 1 mol L^−1^). The resulting equilibrium constant was then used according to the following equation:^99^21
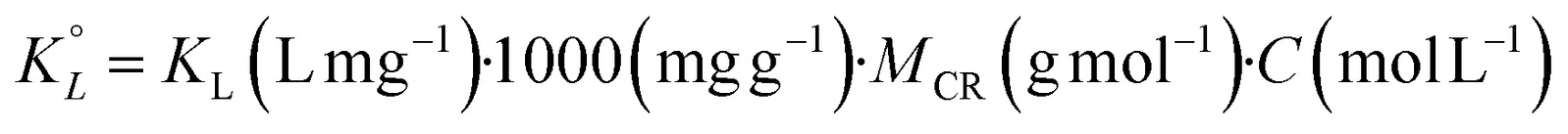
where *M*_CR_ is the molar mass of CR (696.665 g mol^−1^), and the factor 1000 is used to convert grams to milligrams. The plot of 
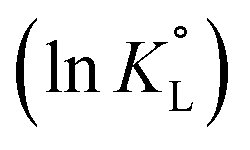
*versus* 1/*T* was constructed and the slope and intercept were used to calculate Δ*H*°(kJ mol^−1^) and Δ*S*°(J mol^−1^), respectively (Table 7).

**Table 7:** Thermodynamic parameters for the adsorption of CR onto MgO NPs

Adsorbent	*T* (K)	Δ*G*° (kJ mol^−1^)	Δ*H*° (kJ mol^−1^)	Δ*S*° (J mol^−1^ K^−1^)
MgO NPs	293	−31.164	−27.205	13.503
	303	−31.299		
	313	−31.434		

The results showed that the adsorption efficiency decreased when the temperature increased from 293 to 313 K, highlighting the importance of energy and entropy in understanding adsorption kinetics.

The negative values of Gibbs free energy (Δ*G*°) at all studied temperatures indicate that the adsorption of CR onto MgO NPs is spontaneous^108^ and thermodynamically favorable. Moreover, the slight increase in the negativity of Δ*G*° with temperature suggests that the spontaneity of the process is maintained within the investigated temperature range.^108^ This behavior is consistent with previous studies reporting spontaneous adsorption processes for dye removal systems.^62,67^

The negative value of enthalpy change (Δ*H*° = −27.205 kJ mol^−1^) indicates that the adsorption process is exothermic. This implies that the adsorption capacity decreases with increasing temperature, which is attributed to the weakening of interactions between CR molecules and the active sites of MgO NPs, as well as the enhanced desorption tendency at higher temperatures. Similar exothermic behavior has been reported in related adsorption systems.^62,67^,^109^ The positive nature of entropy value (Δ*S*° = 13.503 J mol^−1^ K^−1^) suggests an increase in randomness at the solid–solution interface during the adsorption process. This can be attributed to structural changes at the interface and the displacement of water molecules initially adsorbed on the surface, leading to higher disorder in the system. Such behavior has been previously reported in dye adsorption studies, where increased entropy reflects enhanced affinity between the adsorbate and adsorbent surface.^62,67,109^ The positive entropy nature (Δ*S*° > 0) indicates increased disorder during adsorption. This is mainly due to the release of structured water molecules from both the dye and MgO surface when adsorption occurs. The resulting gain in freedom of these molecules outweighs the ordering caused by dye binding, leading to an overall increase in entropy.

Overall, the thermodynamic parameters indicate that the adsorption of CR onto MgO NPs is a spontaneous and exothermic process accompanied by an increase in randomness at the interface, consistent with a favorable adsorption process. These macroscopic thermodynamic trends are in excellent agreement with the microscopic DFT/MD observations, which show strong but energetically favorable binding between CR sulfonate groups and MgO surface sites, coupled with reorganization of interfacial water.

## 4. Computational study using DFT

### 4.1. Application of computational tools and techniques

#### 4.1.1. Density Functional Theory framework

Density Functional Theory (DFT) calculations were performed to investigate the electronic structure and reactivity of Congo Red (CR) molecules. All quantum chemical computations were carried out using the Gaussian 09W software package. The molecular geometry of CR was fully optimized using the hybrid B3LYP functional in combination with the 6-31G(d,p) basis set, which is widely employed for organic systems due to its good balance between accuracy and computational cost. To simulate real-world aqueous processing conditions accurately, all structural optimization protocols were executed within a self-consistent reaction field (SCRF) using the Polarizable Continuum Model (PCM) for water solvent configurations. The standard convergence thresholds were strictly configured to a maximum force criteria of 0.00045 a.u. and a displacement threshold of 0.0018 a.u. To gain deeper insight into the molecular reactivity, frontier molecular orbitals (FMOs), namely the Highest Occupied Molecular Orbital (HOMO) and the Lowest Unoccupied Molecular Orbital (LUMO), were analyzed, frontier molecular orbitals (FMOs), namely the Highest Occupied Molecular Orbital (HOMO) and the Lowest Unoccupied Molecular Orbital (LUMO), were analyzed. The HOMO energy (E_HOMO_) is generally associated with the electron-donating ability of the molecule, whereas the LUMO energy (E_LUMO_) reflects its electron-accepting capacity. The spatial distribution of these orbitals provides valuable information about the potential active sites involved in adsorption interactions.^36,110^

The HOMO–LUMO energy gap (Δ*E*_gap_) was calculated to evaluate the chemical stability and reactivity of the molecule. A relatively small energy gap indicates high chemical reactivity and facilitates electron transfer interactions between the adsorbate and the adsorbent surface, which is favorable for adsorption processes.^111^

In addition, several global reactivity descriptors were derived from the HOMO and LUMO energies using Koopmans' theorem, including ionization potential (*I*), electron affinity (*E*_a_), chemical potential (*µ*), global hardness (*η*), global softness (*S*), electronegativity (*χ*), and electrophilicity index (*ω*), as well as electrodonating (*ω*^−^) and electroaccepting (*ω*^+^) powers.^36,110^ These descriptors provide complementary information regarding the chemical stability, polarity, and reactivity of the molecule, and are widely used to predict adsorption behavior and charge transfer capability.^36,110^ The corresponding equations are summarized in Table 8.

**Table 8:** Equations of global reactivity descriptors

Parameter	Equation
Energy gap (Δ*E*)	Δ*E* = *E*_LUMO_ − *E*_HOMO_
Ionization potential (I)	*I* = −*E*_HOMO_
Electron affinity (Ea)	*E*_a_ = −*E*_LUMO_
Chemical potential (µ)	*µ* = (*E*_HOMO_ + *E*_LUMO_)/2
Global hardness (η)	*η* = (*E*_LUMO_ − *E*_HOMO_)/2
Electronegativity (*χ*)	*χ* = −*µ*
Global softness (S)	*S* = 1/(2*η*)
Maximum charge transfer (ΔNmax)	Δ*N*_max_ = – *µ*/*η*
Electrophilicity index (ω)	*ω* = *µ*^2^/(2*η*)
Electron-accepting power (*ω*^+^)	*ω* ^+^ = (I + 3*E*_a_)^2^/[16 (*I* − *E*_a_)]
Electron-donating power (ω^−^)	*ω* ^−^ = (3*I* + *E*_a_)^2^/[16 (*I* − *E*_a_)]

The equations used to calculate these global reactivity descriptors are summarized in Table 8. These parameters offer useful information regarding the chemical stability and reactivity profile of the molecule.^36,110^

#### 4.1.2. Molecular dynamics simulation

Molecular dynamics (MD) simulations were executed to analyse the adsorption behaviour and interaction energy of the CR dye molecule on the MgO NPs (100) surface. The simulations were performed using the Adsorption Locator module embedded within Materials Studio 2020 (BIOVIA), which operates based on a Monte Carlo (MC) sampling algorithm. This tool systematically explores the potential energy surface to identify favorable adsorption sites, optimal molecular orientations, and the corresponding binding energies of adsorbate–substrate complexes. In this study, the MgO NPs (100) crystallographic plane was selected as the adsorbent surface due to its thermodynamic stability and relevance in environmental remediation applications. The simulation domain was constructed as a (2 × 2) supercell, with dimensions of 25 × 25 × 40 Å^3^, and periodic boundary conditions (PBC) applied in all directions. A vacuum slab of 20 Å was introduced along the *z*-axis to eliminate interlayer interactions between periodic images.

All molecular geometries, including those of the substrate and adsorbate, were fully optimized using the COMPASS force field, which offers reliable parameterization for both organic and inorganic systems. The purpose of this computational investigation was to identify low-energy adsorption configurations and characterize the preferential binding modes of CR on the MgO surface. Binding energy calculations provided insight into the stability and strength of the adsorption method, contributing to a deeper understanding of dye–oxide interactions on a model surface.

The raw total potential energy minimized for the single periodic supercell block equates to an absolute value of (−4258.7 eV). When scaled strictly to standard macroscopic chemical molar equivalents *via* Avogadro's conversion, this reflects a highly stable binding energy that strongly complements the spontaneous (Δ*G*° <)0 and exothermic Δ*H*° = −27.2 kJ mol^−1^ profiles determined experimentally.The MD-derived interaction distances (2.8–3.2 Å) correspond well to the close contacts inferred from thermodynamic analysis and indicate strong electrostatic and hydrogen bonding between CR functional groups and Mg^2+^/O^2−^ sites on the MgO surface.

### 4.2. A theoretical framework based on DFT and molecular dynamics for probing molecular-level interactions

#### 4.2.1. Evaluation of molecular orbitals and reactivity properties

**Fig. 14:**
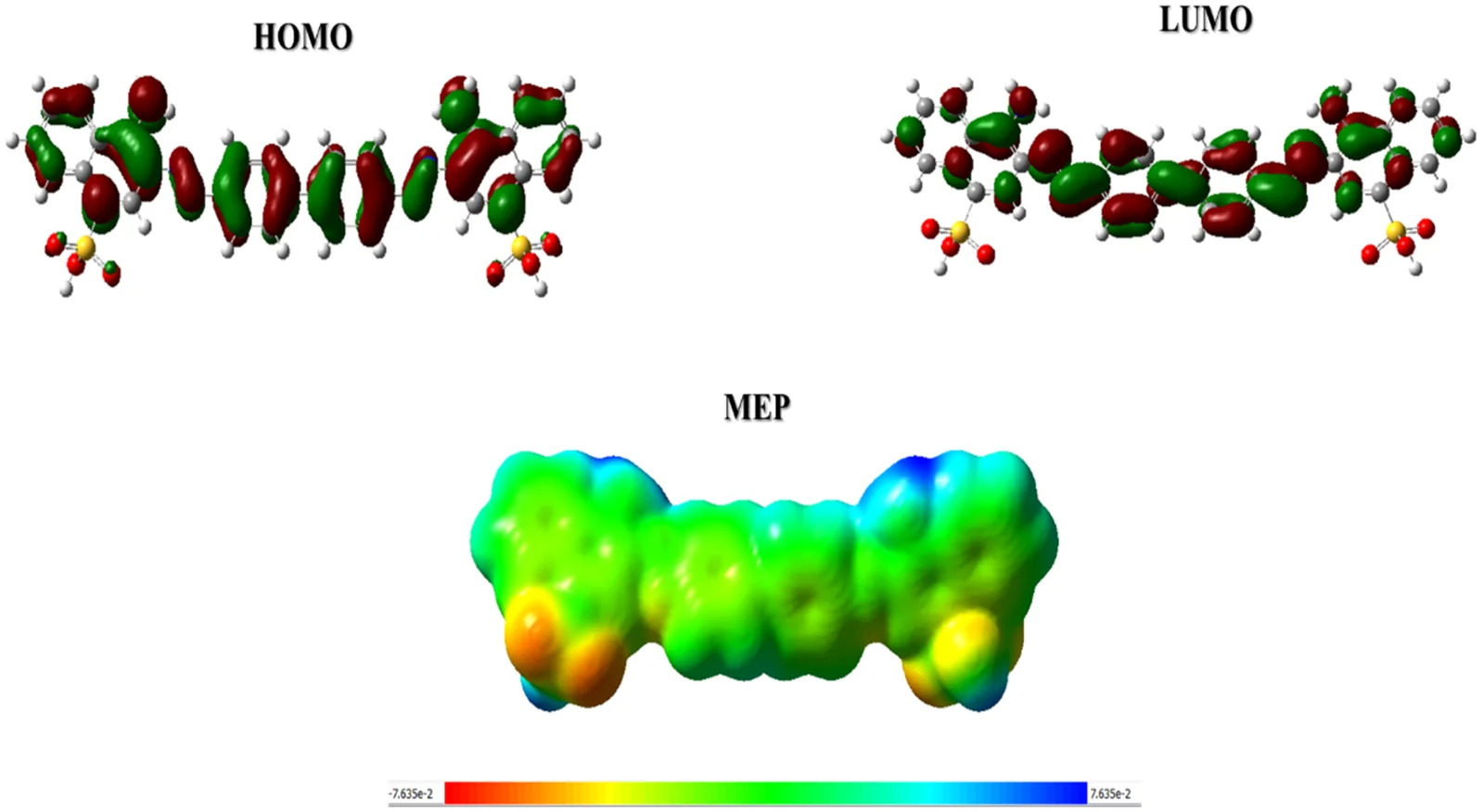
Evaluation of frontier molecular orbitals and electrostatic potential of CR.

The analysis of the frontier molecular orbitals of CR, particularly the HOMO and LUMO, provides valuable insights into its electronic behavior and reactivity toward inorganic surfaces such as MgO. The Highest Occupied Molecular Orbital (HOMO) exhibits strong delocalization across the entire conjugated framework, including the aromatic rings and sulfonate groups (–SO_3_^−^), indicating a high electron density in these regions. This configuration suggests that CR acts as an effective electron donor, capable of establishing interactions with electron-accepting sites, such as surface cations (Mg^2+^) or electrophilic functional groups. The HOMO energy was calculated to be −0.20449 a.u., while the energy of the Lowest Unoccupied Molecular Orbital (LUMO) was −0.10045 a.u., resulting in a HOMO–LUMO gap of 0.10404 a.u., equivalent to approximately (≈2.83 eV). Fig. 14 highlights this electronic distribution, showing the localization of the frontier orbitals and the electrostatic potential around the functional groups of CR. This relatively small energy gap reflects a high electronic reactivity and facilitates adsorption mechanisms involving charge transfer. Furthermore, the LUMO is primarily localized over the central aromatic chain and partially extends toward the terminal rings, indicating the molecule's capacity to accept electrons through π* electronic transitions. This spatial distribution highlights the key role of the conjugated aromatic system in donor–acceptor interactions and suggests a strong affinity for surfaces enriched with lone electron pairs, such as MgO.

The combined analysis of HOMO and LUMO reveals a coherent electron delocalization consistent with the highly conjugated nature of Congo Red, explaining its efficient interaction with adsorptive surfaces. These orbital properties not only contribute to the dye's adsorption performance but also underscore its potential as an active agent in photodegradation, optical sensitization, and pollutant remediation by sorption processes.

These electronic features are consistent with the adsorption energies derived from the Dubinin–Radushkevich model (8.89–18.55 kJ mol^−1^), which indicates a combination of physical and chemical adsorption. The electron-rich HOMO and negatively charged regions identified in the MEP map correspond to sulfonate groups that interact strongly with Mg^2+^ sites, supporting the multi-step adsorption mechanism suggested by the kinetic and thermodynamic studies.

The electrostatic potential (ESP) map projected onto the total electron density of the Congo Red molecule highlights the spatial distribution of surface charges and reveals key chemically active regions. The red and orange areas correspond to zones of high negative electrostatic potential, predominantly located around the sulfonate groups (–SO_3_^−^), indicating a high electron density. These regions represent nucleophilic sites that are likely to interact preferentially with electrophilic centers, such as Mg^2+^ ions on the MgO surface. In contrast, blue regions reflect areas of positive potential, typically associated with electron-deficient zones such as hydrogen atoms or less polar functional groups. The dominant green coloration across the molecular surface indicates a moderate and delocalized potential, consistent with the π-electron delocalization along the conjugated aromatic backbone of the molecule. This heterogeneous charge distribution confirms the high electronic reactivity of CR and its ability to engage in electrostatic interactions, hydrogen bonding, and surface complexation, thereby promoting effective adsorption onto inorganic surfaces such as MgO.

The reactivity parameters calculated from the EHOMO and ELUMO indicate that the studied molecule exhibits a low energy gap, reflecting high chemical reactivity and reduced electronic stability. The ionization potential and *E*_a_ reveal the molecule's ease of donating and accepting electrons, characteristic of systems involved in charge-transfer processes. The negative chemical potential highlights a strong tendency to attract electrons, supported by a moderate electronegativity (*χ*). The calculated reactivity parameters of Congo Red in water, including the HOMO–LUMO energies, chemical potential, hardness, softness, and electrophilicity indices, are summarized in Table 9. The molecule also displays low chemical hardness (*η*) and high softness (*S*), indicating high polarizability and a strong propensity to interact with reactive species. This quantum chemical profile provides a definitive molecular-level justification for the experimental pH-dependent trends observed in Section 3.2.4. The high negative electrostatic potential concentrated on the sulfonate groups SO_3_^−^, visualized as the prominent red/orange nucleophilic regions in the MEP map Fig. 14, acts as the primary energetic driver for surface complexation. At the optimal experimental pH of 7.36, the MgO surface is strongly protonated as Mg–OH_2_^+^ species. The low chemical hardness (*η* = 0.05202 eV and high softness (*S* = 9.610 eV of Congo Red signify high polarizability, which dramatically accelerates the frontier molecular orbital interaction. This enables rapid charge transfer from the electron-rich HOMO of the dye's sulfonate groups directly into the vacant d-orbitals of the electrophilic Mg^2+^ surface sites. Conversely, when the solution pH transitions to an alkaline regime (pH > 10.42), the reversal of the adsorbent's surface charge to a deprotonated MgO state induces a severe quantum mechanical repulsion against these identical electron-rich sulfonate regions, explaining the corresponding drop in experimental adsorption capacity.

**Table 9:** Evaluation of quantum chemical descriptors of Congo Red in water

Symbol	Value (eV)	Symbol	Value (eV)
Δ*E*_gap_	0.10404	*Δ*_Nmax_	5.863
*E*_a_	0.10045	*Ω*	0.8946
*I*	0.20449	*ω* ^+^	0.1537
*µ*	−0.30494	*ω* ^−^	0.3061
*η*	0.05202	*S*	9.610
*χ*	0.30494		

The electrophilicity index, along with the electron-accepting power (*ω*^+^) and electron-donating power (ω^−^), suggests a dual behavior, with a slightly higher tendency toward electron donation. Finally, the maximum number of electrons that can be accepted (Δ*N*_max_) confirms the molecule's capacity to accommodate significant electron transfer before reaching equilibrium, making it a potential candidate for adsorption applications on electron-rich or polarizable surfaces such as metal oxides.

#### 4.2.2. Molecular dynamics simulation of adsorption mechanisms

The molecular dynamics simulation (MDS) results indicate a highly favorable interaction for both the CR molecule and the MgO NPs (100) surface. The very negative adsorption energy suggests a strong and stable chemisorption process, highlighting a significant affinity of the dye for the MgO NPs surface. This is further supported by the system's low total energy, which confirms the thermodynamic stability of the adsorbate–substrate complex.

**Fig. 15:**
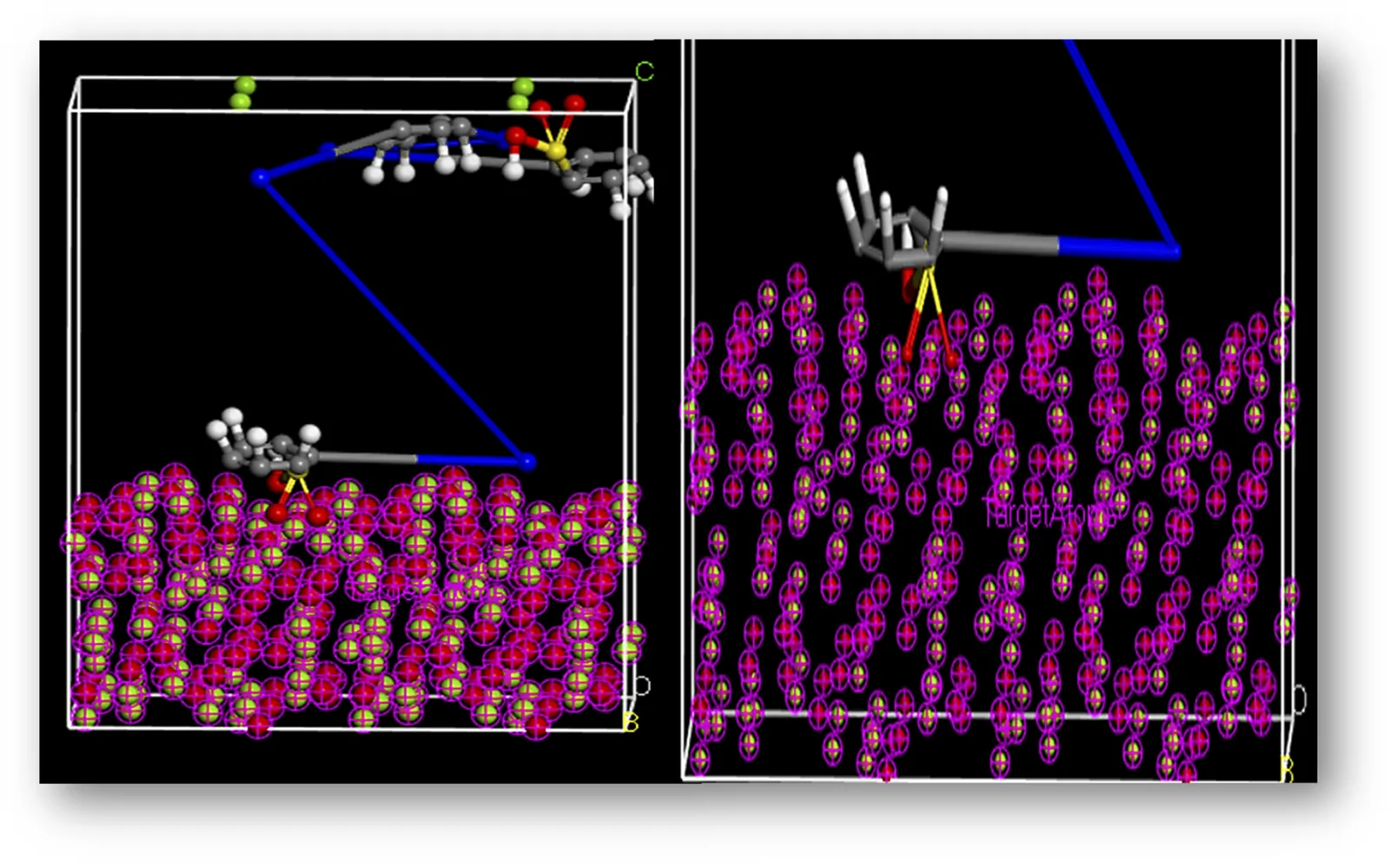
Visualization of multiple stable adsorption modes of CR on MgO (100) *via* the adsorption locator tool.

The relatively high deformation energy implies that the CR molecule undergoes notable structural adjustments to conform to the adsorption sites, reflecting its conformational flexibility. The minimum interaction distance between the Congo Red and the MgO surface ranges from 2.8 to 3.2 Å, which is characteristic of strong non-covalent interactions such as hydrogen bonding and van der Waals forces, confirming the molecule's close proximity and strong anchoring to the surface. Fig. 15 clearly visualizes the multiple stable adsorption modes of CR on the MgO(100) surface, showing the specific interactions between the functional groups of the dye and the active Mg^2+^ and O^2−^ surface sites. The adsorption energies, minimum interaction distances, and deformation energies of Congo Red on the MgO(100) surface obtained from molecular dynamics simulations are summarized in Table 10. The strong negative adsorption energy and close contact distances observed in MD simulations explain the exothermic adsorption process measured experimentally (Δ*H*° = −27.2 kJ mol^−1^) and the spontaneous nature of adsorption (Δ*G*° < 0). The MD results also support the multi-step adsorption mechanism inferred from kinetic and isotherm studies, including both surface interactions and intra-particle diffusion.

**Table 10:** MDS-generated results for the adsorption process of CR onto MgO

Parameter	Value (eV)	Value (molar equivalent kJ mol^−1^)	
Total energy	−4305.714 eV	−415,431.1 kJ mol^−1^	
Adsorption energy	−4258.729 eV	−410,904.3 kJ mol^−1^	
Rigid adsorption energy	−5879.493 eV	−567,272.7 kJ mol^−1^	
Deformation energy	1620.764 eV	156 373.1 kJ mol^−1^	
CR (dEad/dNi)	−4258.729 eV	−410,904.3 kJ mol^−1^	

The thermodynamic analysis revealed negative values of Δ*G*°, confirming the spontaneous nature of the adsorption process, while the negative Δ*H*° indicated an exothermic process.

These findings are strongly supported by the DFT results. The presence of electron-rich regions (HOMO and MEP) localized on the sulfonate groups explains the strong electrostatic attraction between CR molecules and Mg^2+^ surface sites. Such interactions typically release energy, which is consistent with the exothermic behavior observed experimentally.

Furthermore, the positive entropy change (Δ*S*°) can be explained by the displacement of water molecules and the reorganization of dye molecules at the interface, leading to increased disorder. This behavior is consistent with adsorption processes involving electrostatic interactions and surface complexation.

The MD simulations further confirm this interpretation by demonstrating stable adsorption configurations and significant interaction energies, indicating that the adsorption process is both thermodynamically favorable and structurally stable.

The DFT and MD results provide a molecular-level explanation for the experimental adsorption behavior. The electron-rich HOMO and sulfonate groups of Congo Red correspond to the strong interactions with Mg^2+^ sites, consistent with the exothermic and spontaneous adsorption observed in thermodynamic studies (Δ*H*° < 0, Δ*G*° < 0). The MD-derived adsorption energies and interaction distances support the multi-step adsorption mechanism inferred from kinetic and isotherm analyses, confirming both surface adsorption and intra-particle diffusion. Overall, the computational findings quantitatively validate the experimental observations, linking molecular reactivity and structural interactions to the high adsorption capacity and stability of CR on MgO NPs. This tight consistency between theory and experiment strengthens the reliability of the proposed adsorption mechanism and the suitability of the green-synthesized MgO NPs for practical applications.

## 5. Reusability of MgO NPs

**Fig. 16:**
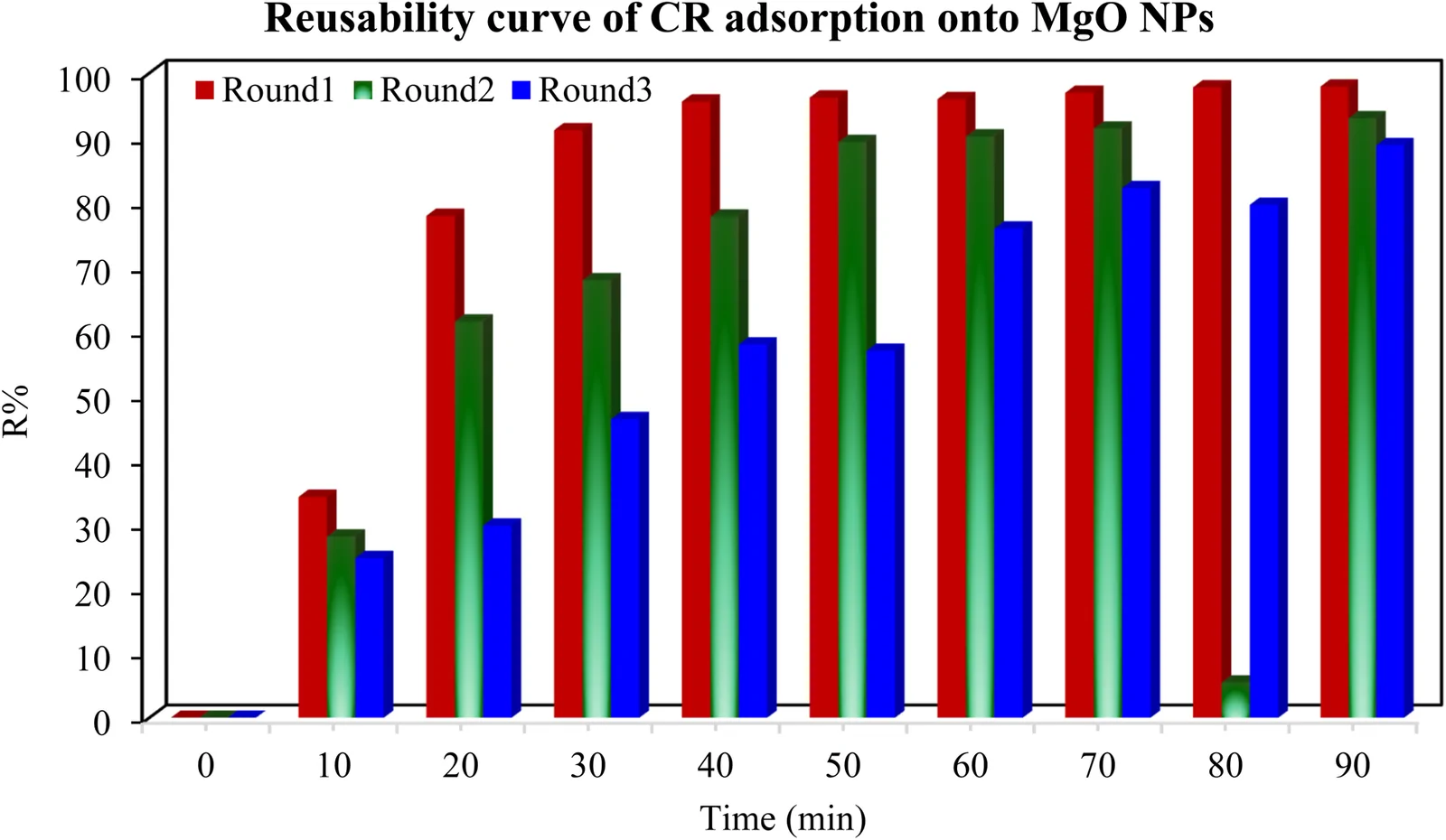
Reusability curve of CR adsorption onto MgO NPs.

Recyclability is an important consideration for the long-term and cost-effective use of adsorbents. To determine the reusability of MgO NPs, their adsorption capacity was measured during three adsorption–desorption cycles. As illustrated in Fig. 16.

The reusability curve of CR adsorption onto MgO NPs presented by Fig. 16 shows that the adsorbent was effectively recycled three times while retaining a high efficiency. Even after the third cycle, it preserved 88.83% of its dye removal capability from the aqueous solution, demonstrating its potential for continued application in wastewater treatment. This moderate loss of performance suggests that, in real systems, periodic regeneration or partial replacement would be required, but the material remains promising for repeated usage in batch or semi-continuous operations.

## 6. Conclusions

In conclusion, magnesium oxide nanoparticles were successfully synthesized *via* a green and sustainable method using olive pomace as a natural reducing and stabilizing agent. Characterization confirmed their crystalline structure nanoscale crystallite size, and porous morphology, suitable for efficient adsorption. The nanoparticles demonstrated remarkable capability for removing Congo Red dye from aqueous solutions, driven by favorable surface interactions, electrostatic attractions, and spontaneous adsorption processes. Thermodynamic and computational studies further supported the stability and efficiency of the adsorption mechanism. The integration of experimental adsorption studies with DFT and MD simulations provided a coherent, molecular-level picture of the interaction between CR and MgO surfaces, explaining the mixed physisorption–chemisorption mechanism and the strong temperature and pH dependencies.

These results highlight the potential of green-synthesized MgO nanoparticles as eco-friendly and sustainable adsorbents for the treatment of dye-contaminated wastewater, providing a promising approach for environmental remediation.

## Author contributions

Conceptualization, S. M., M. Z., A. A. A. and A. A.; methodology, S. M., M. Z. and H. T.; software, Z. L. and O. R. B.; validation, S. M., M. Z., N. H. and S. C.; formal analysis, S. M., M. Z. and H. T.; investigation, S. M., M. Z., H. T. and Z. L.; resources, M. K., M. L. and A. F.; data curation, S. M. and M. Z.; writing – original draft preparation, S. M. and M. Z.; writing—review and editing, A. A. A., J. Z. and A. A.; visualization, F. F. and M. H. R.; supervision, A. A. A. and A. A.; project administration, A. A. A.; funding acquisition, A. A. A. All authors have read and agreed to the published version of the manuscript.

## Conflicts of interest

The authors declare no conflicts of interest.

## Data Availability

The original contributions presented in this study are included in the article. Further inquiries can be directed to the corresponding authors.
